# Identification of Ellagic Acid Rhamnoside as a Bioactive Component of a Complex Botanical Extract with Anti-biofilm Activity

**DOI:** 10.3389/fmicb.2017.00496

**Published:** 2017-03-23

**Authors:** Benjamin M. Fontaine, Kate Nelson, James T. Lyles, Parth B. Jariwala, Jennifer M. García-Rodriguez, Cassandra L. Quave, Emily E. Weinert

**Affiliations:** ^1^Department of Chemistry, Emory UniversityAtlanta, GA, USA; ^2^Department of Dermatology, Emory University School of MedicineAtlanta, GA, USA; ^3^Center for the Study of Human Health, Emory UniversityAtlanta, GA, USA

**Keywords:** biofilm, *Staphylococcus aureus*, natural products, ellagic acid, *Rubus ulmifolius*

## Abstract

*Staphylococcus aureus* is a leading cause of hospital-acquired infections. It is listed among the top “serious threats” to human health in the USA, due in large part to rising rates of resistance. Many *S. aureus* infections are recalcitrant to antibiotic therapy due to their ability to form a biofilm, which acts not only as a physical barrier to antibiotics and the immune system, but results in differences in metabolism that further restricts antibiotic efficacy. Development of a modular strategy to synthesize a library of phenolic glycosides allowed for bioactivity testing and identification of anti-biofilm compounds within an extract of the elmleaf blackberry (*Rubus ulmifolius*). Two ellagic acid (EA) derivatives, EA xyloside and EA rhamnoside, have been identified as components of the *Rubus* extract. In addition, EA rhamnoside has been identified as an inhibitor of biofilm formation, with activity comparable to the complex extract 220D-F2 (composed of a mixture of EA glycosides), and confirmed by confocal laser scanning microscopy analyses.

## Introduction

While bacteria play essential roles in many aspects of human health, as evidenced by the growing body of work on the human microbiome (Kostic et al., 2013; Sommer and Backhed, 2013), bacterial infections can wreak havoc, particularly if the infectious microbes are antibiotic resistant. Currently within the United States, nearly two million people develop hospital-acquired infections each year, the majority of which are antibiotic resistant and result in nearly 100,000 deaths. In addition, antibiotic resistant infections cost the United States between 21 and 34 million dollars each year, resulting in a financial strain on the health care system [Boucher et al., 2009; Roberts et al., 2009; Infectious Diseases Society of America (IDSA) et al., 2011]. Development of new and effective antibiotic treatments, including methods to target alternative pathways, like virulence and pathogenesis (Bodini et al., 2009; Harjai et al., 2010; Cech et al., 2012; Quave et al., 2012; Sully et al., 2014; Talekar et al., 2014), may provide novel methods to treat disease.

Formation of biofilms on native host tissues and indwelling medical devices leads to microbial infections that are recalcitrant to antimicrobials, even in the absence of issues related to acquired resistance. Biofilms increase cross-species gene transfer, lead to expression of more virulent phenotypes, and result in a much higher cell density (10^11^ CFU/mL) than their planktonic counterparts (10^8^ CFU/mL) (Thomas et al., 2006). In addition, the presence of a foreign body decreases the minimal infecting dose of the Gram-positive pathogen *Staphylococcus aureus* >100,000-fold (Zimmerli et al., 1982) and increases the chances of a biofilm infection for individuals with such devices, typically requiring a two-stage treatment involving removal of all foreign material/debridement of infected tissue combined with aggressive antimicrobial treatment (Brause, 2005; Toms et al., 2006).

Biofilms are complex, three-dimensional bacterial communities that can lead to longer hospital stays, recurrent infection, and increased fatalities in the most recalcitrant infections [Boucher et al., 2009; Roberts et al., 2009; Infectious Diseases Society of America (IDSA) et al., 2011]. While there is indeed a pressing need for new antibiotics, there is an equally urgent need to develop drugs that specifically target biofilms to interfere with pathogenesis pathways. Non-biocide biofilm inhibitors could be used to prevent colonization, while avoiding selective pressures for resistance typical of the antibiotics (Agostinho et al., 2009; Aykut et al., 2010; Harth et al., 2010; Jang et al., 2010) and metals (Monteiro et al., 2009; Baldoni et al., 2010; Khalilpour et al., 2010) used in current device coatings. Finally, biofilm inhibitors also could be used as antibiotic adjuvants (Kalan and Wright, 2011) by facilitating antibiotic access to microbial targets, thereby improving efficacy (Wolcott and Dowd, 2010).

*Rubus ulmifolius* Schott. (Rosaceae) is an integral part of the traditional Mediterranean pharmacopeia. Widely recognized as a wild edible plant for its berries, it is also highly valued for treatment of purulent skin and soft tissue infections. It has a chemistry rich in phenolics, many of which are likely the source of its potent antioxidant and antimicrobial activity (Flamini et al., 2002; Panizzi et al., 2002; Luís et al., 2011). Traditional medical use of the roots and leaves was documented in a field survey of south Italian medicinal species (Quave et al., 2008), validated in the lab with initial studies on its anti-staphylococcal activity (Quave et al., 2008), and most recently examined for its potent anti-biofilm properties and ability to improve antibiotic efficacy in the treatment of staphylococcal (Quave et al., 2012) and pneumococcal (Talekar et al., 2014) biofilms. Importantly, further work on this natural product composition could have great implications for future treatment of biofilm-associated infections in the clinical setting.

Previously, anti-biofilm activity in *S. aureus* was used to guide fractionation of roots of the elmleaf blackberry, or *R. ulmifolius*, to isolate an ellagic acid (EA) derivative-rich fraction (referred to as 220D-F2) (Quave et al., 2012). Importantly, the extract produced dose-dependent inhibition of biofilm formation that was conserved across all *S. aureus* clonal lineages, including clinical methicillin-resistant *Staphylococcus aureus* (MRSA) isolates. When 220D-F2 was used concomitantly with antibiotics from varying functional classes to treat an infected device (intravenous catheter), significant (5 log) improvement in biofilm clearance was observed over treatment with antibiotic alone. Furthermore, its range of bioactivity spans to other Gram-positive pathogens, including *Streptococcus pneumoniae* (Talekar et al., 2014). LC–MS/MS analysis of 220D-F2 revealed the presence of a number of EA glycosides (EAGs), including generic 6-deoxypyranose, 5-deoxypyranose, and/or furanose derivatives, suggesting that one or more of these compounds may be responsible for the anti-biofilm activity of the *Rubus* extract. However, MS could not ascertain the identity of the sugars, the anomeric configuration, or the site of glycosylation, and further sub-fractionation has yet to yield information regarding the molecular structure(s). Therefore, a panel of EAGs and analogs have been synthesized and tested to determine if these contribute to the anti-biofilm activity of *R. ulmifolius* extracts, as well as the structural requirements for anti-biofilm activity. These studies will aid in identification of novel anti-biofilm compounds that potentially can be used to inhibit medically relevant biofilms and as adjuvants to treat bacterial infections.

## Materials and Methods

### 220D-F2 Preparation and Analysis

Extract 220D-F2 was prepared from wild harvested samples of *R. ulmifolius* and checked for batch-to-batch reproducibility by HPLC as described (Quave et al., 2012). The presence of EAGs in 220D-F2 was examined by liquid chromatography–Fourier transform mass spectrometry (LC–FTMS) comparison of 220D-F2 and the EAG standards. The LC–FTMS analysis was performed on a Shimadzu SIL-ACHT and Dionex 3600SD HPLC pump. A 20 μL injection for extracts or 5 μL injection for standards was made onto an Agilent Eclipse XDB-C18 4.6 × 250 mm, 5 μm at ambient temperature. A linear gradient consisting of 0.1% formic acid in acetonitrile (A) and 0.1% formic acid in water (B) at a flow rate of 1 mL/min was used for the chromatographic separations. The initial conditions were 98:2 (A:B) changing to 88:12 (A:B) over 34 min, this ratio was held until 50 min, and then increased to 75:25 (A:B) at 70 min, then to 5:95 (A:B) at 82 min and held for 6 min to flush the column before returning to initial conditions. The HPLC was coupled to a Thermo Scientific LTQ-FT Ultra MS in negative electrospray ionization (ESI) mode. The MS was tuned using EA and all data was acquired in MS^1^ mode scanning from a *m/z* of 100–1000 and data dependent MS^2^ collection on a Thermo Scientific LTQ-FT Ultra MS in negative ESI mode and processed with Thermo Scientific Xcalibur 2.2 SP1.48 software (San Jose, CA, USA). The capillary temperature was 275.0°C, sheath gas was N_2_ at 60, source voltage and current 5.0 kV and 100.0 μA, and the capillary voltage -41.00 V.

All of the chemically synthesized phenolic glycosides were subjected to HPLC LC–FTMS analysis using the same conditions described previously (Supplementary Figures S1–S7). The presence of EAGs in 220D-F2 was determined by filtering the extract’s LC–FTMS chromatogram for ions corresponding to the synthesized compound’s experimentally determined negative ion. The retention time of the resulting peaks was compared to the retention time experimentally determined for the synthesized compounds. Additionally, the MS^1^ and MS^2^ for the 220D-F2 peaks were compared to that of the synthesized compounds. Peaks from 220D-F2 which had corresponding retention times, parent ions, and MS^2^ fragmentation patterns were identified as the indicated compounds.

### Growth and Biofilm Inhibition Assays

All test compounds and the 220D-F2 botanical extract control were examined for growth inhibitory and biofilm inhibitory activity following established methods. A well-characterized methicillin-sensitive *Staphylococcus aureus* (MSSA) osteomyelitis isolate (UAMS-1) and its isogenic biofilm-deficient SarA mutant (UAMS-929) were used in these studies. The SarA mutant was selected for use as a control due to its reduced capacity to form a biofilm, which has been demonstrated to significantly increase susceptibilities to antibiotics *in vivo* (Atwood et al., 2016). For microbiological tests, strains were grown from freezer stock onto tryptic soy agar (TSA) plates, and then overnight cultures grown in tryptic soy broth. All cultures were grown at 37°C. For growth inhibition studies, MIC_50_ and MIC_90_ values (representing the minimum inhibitory concentration for 50 or 90% of the growth control, respectively) were determined following The Clinical Laboratory Standards Institute (CLSI) M100-S23 guidelines for microtiter broth dilution testing (NCCLS, 2001). Briefly, overnight cultures were diluted in cation-adjusted Mueller–Hinton broth (CAMHB) by optical density (OD) to 5 × 10^5^ CFU/mL, and this was confirmed by plate counts. Controls included the vehicle [dimethyl sulfoxide (DMSO)] and antibiotics: ampicillin (MP Biomedical) and vancomycin (Sigma-Aldrich). Two-fold serial dilutions were performed on a 96-well plate to achieve a test range of 4–512 μg/mL for test compounds and 0.5–64 μg/mL for antibiotics. Plates were incubated at 37°C for 18 h. Plates were read at an OD of 600 nm in a Cytation 3 multimode plate reader (Biotek) at 0 and 18 h post-inoculation. The percent inhibition of growth was calculated as described (Quave et al., 2012).

Growth curve experiments were also conducted, with OD_600__nm_ readings taken at 0, 3, 5, 8, 10, 12, and 18 h post-inoculation. The number of colony forming units per mL of culture was measured by diluting and plating the culture on TSA at 18 h post-treatment and counting colonies following 22 h of incubation at 37°C.

Inhibition of biofilm formation was assessed in a static 96-well plate model with human plasma. Briefly, 20% human plasma diluted in carbonate buffer was added to the biofilm media [tryptic soy broth supplemented with 3.0% NaCl (wt/vol) and 0.5% dextrose (wt/vol)] to reach a final concentration of 2% human plasma in the media. Following addition of the test compounds and inoculation with UAMS-1, plates were incubated at 37°C for 22 h. Planktonic cells were then gently aspirated and the wells rinsed twice with phosphate-buffered saline (PBS) to remove non-adherent cells. Adherent biofilms were fixed with 200 μL of 100% ethanol prior to staining for 15 min with 50 μL of 2% (wt/vol) crystal violet in 20% ethanol (Hardy Diagnostics). The stain was washed with tap water, then after drying, 100 μL of 10% of [2.5% Tween 80_(aq)_] in ethanol (EtOH) was added to wells and incubated for 15 min. The eluate (20 μL) was transferred to a new plate containing 180 μL PBS/well and the OD_595__nm_ measured by plate reader. The minimum biofilm-inhibiting concentration (MBIC) was defined as the lowest concentration of extract in which biofilm formation was limited to a level ≥90% (for MBIC_90_) or ≥50% (for MBIC_50_) by comparison to the vehicle-treated control (UAMS-1) strain.

Follow-up assays using the two-dimensional checkerboard method (Farha et al., 2013) were conducted to determine any synergistic activities of the compounds found in extract 220D-F2 (EA, EA rhamnoside, and EA xyloside). The fractional inhibitory concentration (FIC) index for each compound was calculated as the drug in presence of co-drug for a well showing <50% biofilm formation (measured by OD_595__nm_ of crystal violet eluate), divided by the MBIC_50_ for that drug. The FIC index is the sum of the two FICs and interactions with a FIC index ≤0.5 were considered synergistic and FIC of 1 additive.

### Microscopy

Parallel to the above described biofilm assay, biofilm architecture was assessed by confocal laser scanning microscopy (CLSM). Briefly, biofilms were formed as described above (including treatment and control groups). After 20 h, the well contents were aspirated and the wells gently washed three times with 0.85% (wt/vol) NaCl. The adherent biofilm was then stained with LIVE/DEAD stain (Invitrogen) at room temperature in the dark for 15 min, following manufacturer’s protocol. Then CLSM images were collected using an Olympus FluoView 1000 confocal scanning system and total internal reflection fluorescence (TIRF) inverted microscope. SYTO 9 fluorescence was detected by excitation at 488 nm and emission at 527 nm, fluo-3 bandpass filter. Propidium iodide fluorescence was detected by excitation/emission at 543/612 nm, Texas Red bandpass filter. All z-sections were collected at 4-μm intervals using a 10 × objective lens. A 1.27 × 1.27 mm section of biofilm was selected from the center of the well for each image. Image acquisition and processing was performed using Olympus Fluoview software. Identical acquisition settings were employed for all samples.

### Statistical Analysis

All assays performed were analyzed using a two-tailed Student’s *t*-test with unequal variance as calculated by Microsoft Excel 2010. DMSO treated (vehicle control) cultures were used as a vehicle control and were compared to those treated with extract for all statistical analyses. *P*-values <0.05 were considered statistically significant. All assays and other experiments were performed in triplicate or quadruplicate.

### Chemical Synthesis

All reactions were performed using dry solvents in flame-dried glassware under a nitrogen atmosphere with magnetic stirring, unless otherwise noted. Reaction solvents were dried over 4 Å molecular sieves, according to a published procedure (Bradley et al., 2010). EA was purchased from Sigma-Aldrich and stored in a vacuum desiccator over Drierite^TM^ and phosphorous pentoxide. Phenol and catechol were obtained from Spectrum Chemical (New Brunswick, NJ, USA) and TCI American (Portland, OR, USA). L-rhamnose was purchased from Sigma-Aldrich; D-mannose and D-xylose were obtained from Chem-Impex International Inc. (Wood Dale, IL, USA). All commercially sourced chemicals and reagents were used as received. Thin layer chromatography (TLC) was performed on alumina plates coated with silica gel 60 F_254_ and visualized under UV light or by staining with basic KMnO_4_ solution. Silica gel 60 (40–63 μm) was used for flash column chromatography. ^1^H and ^13^C NMR (nuclear magnetic resonance) spectra were recorded on UNITY Plus 600, INOVA 400, and Mercury 300 spectrometers. Chemical shifts are reported in ppm and referenced to the residual solvent signal (for ^1^H NMR: CDCl_3_ = 7.26 ppm, CD_3_OD = 3.31 ppm, DMSO-d6 = 2.50 ppm; for ^13^C NMR: CDCl_3_ = 77.16 ppm, CD_3_OD = 49.00 ppm, DMSO-d6 = 39.52 ppm). High-resolution mass spectra were obtained using a Thermo LTQ FTMS.

#### 1,2,3,4-Tetra-*O*-Acetyl α-L-Rhamnopyranoside (10:1 α:β)

The compound was synthesized from L-rhamnose according to a published procedure and the spectra match previously published data (Donahue and Johnston, 2006) (8.60 g in 85% yield).

#### 1,2,3,4,5-Penta-*O*-Acetyl α-D-Mannopyranoside (6:1 α:β)

The compound was synthesized from D-mannose according to a published procedure and the spectra match previously published data (Yu and Kizhakkedathu, 2010) (1.75 g in 84% yield).

#### 1,2,3,4-Tetra-*O*-Acetyl β-D-Xylopyranoside (4:1 β:α)

The compound was synthesized from D-xylose according to a published procedure and the spectra match previously published data (Camponovo et al., 2009) (5.53 g in 87% yield).

#### General Glycosylation Method (Jacobsson et al., 2006)

The appropriate phenol (1 eq) was dissolved in dichloromethane (DCM) containing 4 Å MS. The per-*O*-acetyl glycosyl donor (1 eq) was then added as a solution in dry DCM, followed by dropwise addition of BF_3_-OEt_2_ (1 eq). The reaction was stirred at room temperature (rt) and monitored by silica gel TLC. The reaction was quenched with saturated aqueous NaHCO_3_ and extracted with CHCl_3_. The combined organic fractions were washed with water and brine, dried over Na_2_SO_4_, filtered, and concentrated *in vacuo*. The residue was purified by silica gel flash column chromatography and all solvent was removed *in vacuo*.

#### General De-*O*-Acetylation Method (Parker et al., 2012)

The per-*O*-acetyl glycoside (1 eq) was dissolved in MeOH and Et_3_N (1 eq) was added. The solution was stirred at rt and monitored by silica gel TLC. Upon completion of the reaction, the volatiles were removed by azeotropic distillation with toluene *in vacuo*.

#### 1-*O*-Phenyl 2,3,4-Tri-*O*-Acetyl α-L-Rhamnopyranoside (**11**)

The compound was synthesized according to the **general glycosylation method**. The residue was purified by silica gel flash column chromatography (325:1 CHCl_3_/MeOH) to afford 136 mg as white needles in 24% yield. ^1^H NMR (400 MHz, CDCl_3_) δ 7.34–7.28 (m, 2H), 7.10–7.07 (m, 2H), 7.06–7.02 (m, 1H), 5.53 (dd, *J* = 10.1, 3.5 Hz, 1H), 5.47 (d, *J* = 1.8 Hz, 1H), 5.44 (dd, *J* = 3.5, 1.9 Hz, 1H), 5.16 (t, *J* = 10.0 Hz, 1H), 4.01 (dq, *J* = 9.9, 6.2 Hz, 1H), 2.20 (s, 3H), 2.07 (s, 3H), 2.04 (s, 3H), 1.21 (d, *J* = 6.3 Hz, 3H). ^13^C NMR (100 MHz, CDCl_3_) δ 170.17, 170.13, 170.11, 156.04, 129.73, 122.85, 116.53, 95.86, 71.18, 69.92, 69.10, 67.26, 21.02, 20.92, 20.87, 17.58. High resolution mass spectrometry (HRMS) (ESI) *m/z*: [M + Na]^+^ calcd for C_18_H_22_O_8_Na^+^ 389.1207; found 389.1201.

#### Phenyl α-L-Rhamnopyranoside (**8**)

The compound was synthesized according to the **general de-*O*-acetylation method** to afford the desired product as a colorless oil (74 mg, 99% yield). ^1^H NMR (400 MHz, CD_3_OD) δ 7.31–7.25 (m, 2H), 7.08–7.03 (m, 2H), 7.02–6.97 (m, 1H), 5.42 (d, *J* = 1.8 Hz, 1H), 4.00 (dd, *J* = 3.4, 1.8 Hz, 1H), 3.85 (dd, *J* = 9.5, 3.5 Hz, 1H), 3.69–3.60 (m, 1H), 3.46 (t, *J* = 9.5 Hz, 1H), 1.22 (d, *J* = 6.2 Hz, 4H). ^13^C NMR (100 MHz, CD_3_OD) δ 157.84, 130.48, 123.17, 117.51, 99.83, 73.85, 72.25, 72.09, 70.57, 18.01. HRMS (ESI) *m/z*: [M + Na]^+^ calcd for C_12_H_16_O_5_Na^+^ 263.0890; found 263.0885.

#### 1-*O*-(*o*-Hydroxy)Phenyl 2,3,4-Tri-*O*-Acetyl α-L-Rhamnopyranoside (**12**)

The compound was synthesized according to the **general glycosylation method**. The residue was purified by silica gel flash column chromatography (60:1 CHCl_3_/MeOH) to afford 51 mg as white needles in 10% yield. ^1^H NMR (400 MHz, CDCl_3_) δ 7.15 (d, *J* = 8.1 Hz, 1H), 6.97 (d, *J* = 4.0 Hz, 2H), 6.82 (dt, *J* = 8.3, 4.5 Hz, 1H), 6.12 (d, *J* = 4.8 Hz, 1H), 5.51 (dd, *J* = 3.5, 1.9 Hz, 1H), 5.47 (dd, *J* = 9.9, 3.5 Hz, 1H), 5.44 (d, *J* = 1.8 Hz, 1H), 5.18 (t, *J* = 9.9 Hz, 1H), 4.06 (dq, *J* = 9.8, 6.2 Hz, 1H), 2.19 (s, 4H), 2.08 (s, 3H), 2.04 (s, 3H), 1.25 (d, *J* = 6.3 Hz, 3H). ^13^C NMR (100 MHz, CDCl_3_) δ 170.43, 170.15, 170.07, 146.10, 143.61, 123.98, 120.54, 116.27, 115.64, 97.14, 70.80, 69.58, 69.16, 67.66, 20.94, 20.88, 20.83, 17.54. HRMS (ESI) *m/z*: [M + Na]^+^ calcd for C_18_H_22_O_9_Na^+^ 405.1156; found 405.1164.

#### *o*-Hydroxyphenyl α-L-rhamnopyranoside (**9**)

The compound was synthesized according to the **general de-*O*-acetylation method** to afford the desired product as a white solid (34 mg, 99% yield). The spectra match previously published data (De Winter et al., 2013).

#### 1-*O*-Phenyl 2,3,4,5-Tetra-*O*-Acetyl α-D-Mannopyranoside (**13**)

The compound was synthesized according to the **general glycosylation method**. The residue was purified by silica gel flash column chromatography (3:2 hexanes/EtOAc) to afford 260 mg as a white solid in 33% yield. ^1^H NMR (300 MHz, CDCl_3_) δ 7.36–7.28 (m, 2H), 7.13–7.03 (m, 3H), 5.58 (dd, *J* = 10.0, 3.5 Hz, 1H), 5.54 (d, *J* = 1.8 Hz, 1H), 5.46 (dd, *J* = 3.5, 1.9 Hz, 1H), 5.38 (t, *J* = 10.1 Hz, 1H), 4.34–4.25 (m, 1H), 4.15–4.04 (m, 2H), 2.21 (s, 3H), 2.06 (s, 3H), 2.05 (s, 3H), 2.04 (s, 3H). ^13^C NMR (100 MHz, CDCl_3_) δ 170.65, 170.10, 170.05, 169.88, 155.77, 129.77, 123.18, 116.69, 95.99, 69.64, 69.31, 69.09, 66.21, 62.32, 21.01, 20.83, 20.78. HRMS (ESI) *m/z*: [M + Na]^+^ calcd for C_20_H_24_O_10_Na^+^ 447.1262; found 447.1265.

#### Phenyl α-D-Mannopyranoside (**4**)

The compound was synthesized according to the **general de-*O*-acetylation method** to afford the desired product as a colorless oil (25 mg, 54% yield). The spectra match previously published data (Klein et al., 2010).

#### *o*-Hydroxyphenyl α-D-Mannopyranoside (**5**)

The compound was synthesized by glycosylation of catechol with per-*O*-acetyl α-D-mannopyranoside according to the **general glycosylation method**. The crude protected glycoside was then deprotected without prior purification according to the **general de-*O*-acetylation method**. The product was isolated via silica gel flash column chromatography (10:1 EtOAc/MeOH, 1% AcOH) in 43% yield (17 mg). ^1^H NMR (600 MHz, CD_3_OD) δ 7.19 (dd, *J* = 8.1, 1.4 Hz, 1H), 6.90–6.85 (m, 1H), 6.83 (dd, *J* = 8.0, 1.7 Hz, 1H), 6.78–6.73 (m, 1H), 5.40 (d, *J* = 1.8 Hz, 1H), 4.12 (dd, *J* = 3.4, 1.8 Hz, 1H), 3.99 (dd, *J* = 8.7, 3.3 Hz, 1H), 3.79–3.73 (m, 4H). ^13^C NMR (150 MHz, CD_3_OD) δ 148.78, 145.89, 124.43, 120.90, 119.42, 117.29, 101.55, 75.40, 72.33, 71.92, 68.42, 62.67. HRMS (ESI) *m/z*: [M + Na]^+^ calcd for C_12_H_16_O_7_Na^+^ 295.0788; found 295.0788.

#### Phenyl β-D-Xylopyranoside (**6**)

The compound was synthesized by glycosylation of phenol with per-*O*-acetyl β-D-xylopyranoside according to the **general glycosylation method**. The crude protected glycoside was then deprotected without prior purification according to the **general de-*O*-acetylation method**. The product was isolated as a white solid via silica gel flash column chromatography (15:1 EtOAc/MeOH, 1% Et_3_N) in 59% yield (252 mg). The spectra match previously published data (Siegbahn et al., 2015).

#### 2,3,4-Tri-*O*-Acetyl α-L-Rhamnopyranosyl Iodide

The compound was synthesized by iodination of **1,2,3,4-tetra-*O*-acetyl α-L-rhamnopyranoside** according to a published procedure and the spectra match previously published data (Mukhopadhyay et al., 2004) (1.95 g, 81% yield).

#### 2,3,4-Tri-*O*-Acetyl α-D-Xylopyranosyl Iodide

The compound was synthesized by iodination of **1,2,3,4-tetra-*O*-acetyl β-D-xylopyranoside** according to a published procedure and the spectra match previously published data (Mukhopadhyay et al., 2004) (4.02 g, 83% yield).

#### 3,3′,4,4′-Tetrakis-*O*-*tert*-Butyldimethylsilyl Ellagic Acid (**16**)

The compound was synthesized in analogy to a published procedure with some modifications (Kobayashi et al., 2013). EA (2 g, 6.62 mmol), 4-(dimethylamino)pyridine (DMAP) (18.5 mg, 0.199 mmol), and imidazole (2.25 g, 33.1 mmol) were suspended in CH_2_Cl_2_/dimethylformamide (DMF) (30 mL/10 mL). A solution of *t*-butyldimethylsilyl chloride (TBS-Cl) (5 g, 33.1 mmol) in CH_2_Cl_2_ was added and the mixture was stirred at 50°C in the dark for 48 h. The reaction was cooled to rt and quenched with saturated aqueous NH_4_Cl. The aqueous phase was extracted with CH_2_Cl_2_ and the combined organic phases were dried over Na_2_SO_4_, filtered, and concentrated *in vacuo*. TBS-OH was removed by azeotropic distillation with toluene *in vacuo*. The residue was then adsorbed onto silica gel and purified by silica gel flash column chromatography (30:1 hexanes/EtOAc) to obtain the desired compound as a pale yellow solid (2.64 g, 70% yield). ^1^H NMR (400 MHz, CDCl_3_) δ 7.64 (s, 2H), 1.09 (s, 18H), 1.02 (s, 18H), 0.34 (s, 12H), 0.32 (s, 12H). ^13^C NMR (100 MHz, CDCl_3_) δ 159.09, 150.15, 141.21, 140.68, 116.46, 113.69, 110.47, 77.16, 26.15, 26.00, 18.94, 18.86, -3.49, -3.65. HRMS [atmospheric pressure chemical ionization (APCI)] *m/z*: [M + H]^+^ calcd for C_38_H_63_O_8_Si_4_^+^ 759.3595; found 759.3594.

#### 3-*O*-(2′′,3′′,4′′-Tri-*O*-Acetyl α-L-Rhamnopyranosyl)-3′,4,4′-Tris-*O*-*tert*-Butyldimethylsilyl Ellagic Acid **(17)**

The synthesis was based on published glycosylation methodology (Du and Gervay-Hague, 2005). Per-*O*-TBS EA **(16)** (930 mg, 1.23 mmol) was dissolved in CH_2_Cl_2_ (15 mL) containing 4 Å MS. The solution was stirred at rt for 1 h in the dark. A solution of tris(dimethylamino)sulfonium difluorotrimethylsilicate (TASF; 371 mg, 1.35 mmol) in CH_2_Cl_2_ was added dropwise and the mixture was stirred at rt under a dry nitrogen purge for 10 min (to remove gaseous Me_3_Si-F). The deprotection was monitored by silica gel TLC. **2,3,4-Tri-*O*-acetyl α-L-rhamnopyranosyl iodide** (1.9 g, 4.9 mmol) was subsequently added as a solution in CH_2_Cl_2_ and the temperature was gradually increased to 45°C. After 48 h the reaction was cooled to rt and filtered to remove 4 Å and insoluble material. The filtrate was concentrated *in vacuo* and the amber residue was dissolved in CH_2_Cl_2_, adsorbed onto Celite (pre-washed with MeOH), and purified by silica gel flash column chromatography (3:1 hexanes/EtOAc). The desired product was obtained as a pale yellow, glassy solid (145 mg, 13% yield). ^1^H NMR (400 MHz, CDCl_3_) δ 7.65 (s, 2H), 5.75 (dd, *J* = 3.3, 1.8 Hz, 1H), 5.67 (d, *J* = 1.5 Hz, 1H), 5.60 (dd, *J* = 10.2, 3.4 Hz, 1H), 5.19 (t, *J* = 10.1 Hz, 1H), 4.78 (dq, *J* = 12.5, 6.3 Hz, 1H), 2.19 (s, 3H), 2.11 (s, 3H), 2.03 (s, 3H), 1.21 (d, *J* = 6.2 Hz, 3H), 1.09 (s, 9H), 1.02 (s, 9H), 1.01 (s, 9H), 0.35 (s, 3H), 0.35 (s, 3H), 0.34 (s, 6H), 0.32 (s, 6H). ^13^C NMR (100 MHz, CDCl_3_) δ 170.24, 170.06, 169.78, 158.67, 158.56, 151.02, 150.50, 143.40, 141.45, 140.60, 139.60, 116.78, 116.54, 114.44, 113.72, 113.33, 110.30, 99.77, 70.56, 69.31, 68.81, 26.12, 25.98, 25.85, 20.98, 20.96, 20.84, 18.94, 18.86, 18.62, 17.33, -3.49, -3.64, -3.99, -4.05. HRMS (ESI) *m/z*: [M + Na]^+^ calcd for C_44_H_64_O_15_Si_3_Na^+^ 939.3445; found 939.3447.

#### 3-*O*-(2′′,3′′,4′′-Tri-*O*-Acetyl β-D-Xylopyranosyl)-3′,4,4′-Tris-*O*-*tert*-Butyldimethylsilyl Ellagic Acid **(18)**

The synthesis was based on published glycosylation methodology (Du and Gervay-Hague, 2005). Per-*O*-TBS EA **(16)** (1.16 g, 1.53 mmol) was dissolved in CH_2_Cl_2_ (15 mL) containing 4 Å MS. The solution was stirred at rt for 1 h in the dark. A solution of TASF (463 mg, 1.68 mmol) in CH_2_Cl_2_ was added dropwise and the mixture was stirred at rt under a dry nitrogen purge for 10 min (to remove gaseous Me_3_Si-F). The deprotection was monitored by silica gel TLC. Bu_4_NI (565 mg, 1.53 mmol) was subsequently added to the reaction as a solution in CH_2_Cl_2_, followed by addition of **2,3,4-tri-*O*-acetyl β-D-xylopyranosyl iodide** (3.8 g, 6.4 mmol) as a solution in CH_2_Cl_2_. The temperature was gradually increased to 45°C. After 48 h the reaction was cooled to rt and concentrated *in vacuo*. The residue was then cooled in an ice bath and ice-cold EtOAc was added to precipitate Bu_4_NI, which was removed by filtration. The filtrate was concentrated *in vacuo* and the amber residue was dissolved in CH_2_Cl_2_, adsorbed onto Celite (pre-washed with MeOH), and purified by silica gel flash column chromatography (3:1 hexanes/EtOAc). The desired product was obtained as a pale yellow, glassy solid (209 mg, 15% yield). ^1^H NMR (400 MHz, CDCl_3_) δ 7.62 (s, 1H), 7.60 (s, 1H), 5.74 (d, *J* = 5.0 Hz, 1H), 5.30 (dd, *J* = 7.2, 5.0 Hz, 1H), 5.22 (t, *J* = 6.9 Hz, 1H), 5.04–4.98 (m, 1H), 4.41 (dd, *J* = 12.5, 4.2 Hz, 1H), 3.53 (dd, *J* = 12.4, 6.0 Hz, 1H), 2.13 (s, 3H), 2.13 (s, 3H), 2.05 (s, 3H), 1.06 (s, 9H), 1.00 (s, 9H), 0.99 (s, 9H), 0.31 (s, 6H), 0.29 (s, 6H), 0.28 (s, 3H), 0.26 (s, 3H). ^13^C NMR (100 MHz, CDCl_3_) δ 170.03, 169.95, 169.41, 158.65, 151.04, 150.43, 142.63, 141.44, 140.57, 139.39, 116.75, 114.10, 113.69, 113.38, 110.26, 100.03, 69.96, 69.91, 68.57, 62.10, 26.10, 25.95, 25.79, 20.93, 20.91, 20.87, 18.91, 18.84, 18.55, -3.52, -3.66, -4.11, -4.20. HRMS (ESI) *m/z*: [M + Na]^+^ calcd for C_43_H_62_O_15_Si_3_Na^+^ 925.3289; found 925.3281.

#### General Ellagic Acid Glycoside Deprotection Method

Deprotection of TBS ethers was performed in analogy to a published procedure (Jiang and Wang, 2003). The appropriate EAG (1 eq) was dissolved in DMF/H_2_O (10:1 v/v) and K_2_CO_3_ (1.3 eq) was added. The solution was stirred at rt in the dark for 5 h. The mixture was diluted with toluene/MeOH and the pH was adjusted to ∼6 with dilute aqueous AcOH. The solvent was removed *in vacuo* and the residue was subjected to azeotropic distillation with toluene. The resulting de-silylated glycoside was suspended in MeOH/H_2_O (10:1 v/v) and K_2_CO_3_ (1.5 eq) was added to affect deprotection of the sugar moiety. The mixture was stirred at rt in the dark for 48 h. The reaction was then diluted with MeOH/H_2_O and the pH was adjusted to ∼4 by gradual addition of Dowex 50WX8 cation-exchange resin (pre-washed with MeOH/H_2_O). The resin was removed by filtration and washed thoroughly with MeOH/H_2_O. The solvent was removed *in vacuo* and the resulting solid was washed with Et_2_O. The resulting material was then dissolved in 10 mM NH_4_HCO_3_ (pH 7.8) and lyophilized to dryness to afford the desired deprotected EAGs.

#### 3-*O*-α-L-Rhamnopyranosyl Ellagic Acid (**10**)

The compound was prepared according to the **general EAG deprotection method** to afford the desired product as a yellow solid (18 mg, 86% yield). ^1^H NMR (400 MHz, DMSO-d6) δ 7.56 (s, 1H), 7.44 (s, 1H), 5.51 (s, 1H), 4.15 (dq, *J* = 12.6, 6.2 Hz, 1H), 4.05 (dd, *J* = 3.1, 1.7 Hz, 1H), 3.78 (dd, *J* = 9.4, 3.3 Hz, 1H), 3.31 (t, *J* = 9.5 Hz, 1H), 1.07 (d, *J* = 6.2 Hz, 3H). ^13^C NMR (100 MHz, DMSO-d6) δ 159.06, 158.71, 152.30, 148.78, 142.40, 136.61, 136.02, 113.26, 112.33, 111.85, 111.10, 109.68, 102.43, 71.40, 70.54, 70.36, 70.13, 17.71. HRMS (ESI) *m/z*: [M - H+]^-^ calcd for C_20_H_15_O_12_^-^ 447.0569; found 447.0574.

#### 3-*O*-β-D-Xylopyranosyl Ellagic Acid (**7**)

The compound was prepared according to the **general EAG deprotection method** to afford the desired product as a yellow solid (22 mg, 91% yield). ^1^H NMR (400 MHz, DMSO-d6) δ 7.50 (s, 1H), 7.38 (s, 1H), 5.35 (d, *J* = 6.9 Hz, 1H), 3.80 (dd, *J* = 11.4, 5.0 Hz, 1H), 3.47–3.39 (m, 2H), 3.27 (t, *J* = 8.3 Hz, 1H), 3.10 (dd, *J* = 11.4, 9.3 Hz, 1H). ^13^C NMR (100 MHz, DMSO-d6) δ 159.80, 159.20, 151.48, 142.14, 135.78, 135.06, 113.56, 113.06, 112.49, 110.47, 106.64, 102.92, 75.40, 73.16, 69.29, 65.69. HRMS (ESI) *m/z*: [M - H+]^-^ calcd for C_19_H_13_O_12_^-^ 433.0412; found 433.0417.

## Results

**FIGURE 1:**
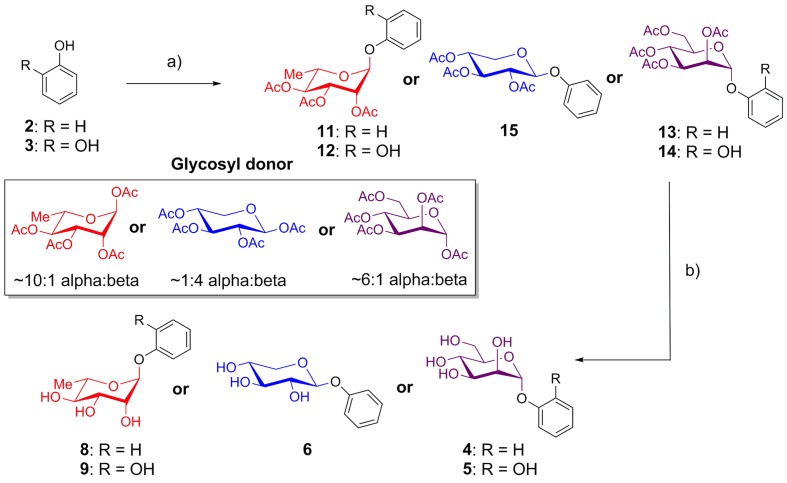
**Synthesis of phenol and catechol glycosides**. (a) BF_3_-OEt_2_, CH_2_Cl_2_, glycosyl donor, room temperature, 10-43%; (b) Et_3_N, MeOH, rt, 99%.

Phenol and catechol were glycosylated using per-*O*-acetyl glycosyl donors (Donahue and Johnston, 2006; Camponovo et al., 2009; Yu and Kizhakkedathu, 2010) in the presence of BF_3_-OEt_2_ (Jacobsson et al., 2006). Reactions with acetates of α-L-rhamnopyranose and α-D-mannopyranose afforded anomerically pure α-glycosides **11–14**, while glycosylation with β-D-xylopyranose provided only the β-glycoside **15**. The resulting protected glycosides were de-*O*-acetylated with triethylamine in methanol (Parker et al., 2012), yielding the desired glycosides of phenol (**4**, **6**, **8**) and catechol (**5**, **9**) (**Figure 1**).

**FIGURE 2:**
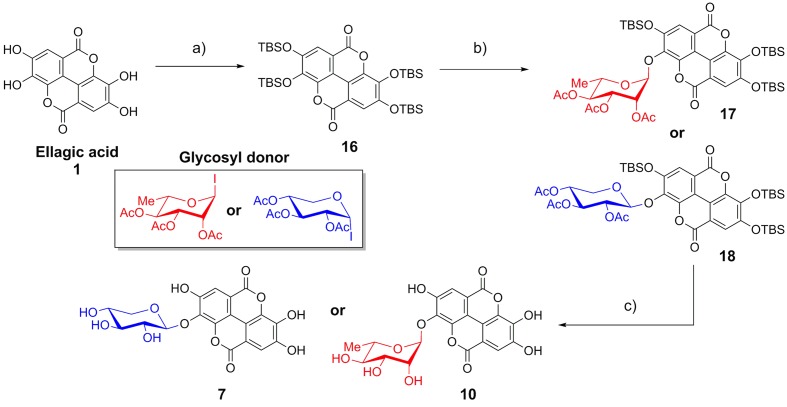
**Synthesis of ellagic acid glycosides**. (a) TBSCl, Im, DMAP, CH_2_Cl_2_/DMF, 50°C, 36 h, 71%; (b) TASF, CH_2_Cl_2_, room temperature, 10 min; then glycosyl donor, Bu_4_NI (xyloside only), reflux, 48 h, 13–15%. (c) (1) K_2_CO_3_, DMF/H_2_O; (2) K_2_CO_3_, MeOH/H_2_O, 86–92%.

As EA is extremely insoluble and quite unreactive, synthesis of EAGs employed per-*O*-acetyl α-glycosyl iodide donors (Gervay and Hadd, 1997; Gervay et al., 1997; Hadd and Gervay, 1999; Bhat and Gervay-Hague, 2001; Du and Gervay-Hague, 2005; Schombs et al., 2010), synthesized by treating per-*O*-acetyl sugars with trimethylsilyl iodide (TMSI), generated *in situ* from iodine and hexamethyldisilane (Mukhopadhyay et al., 2004). To control the regioselectivity of the glycosylation, EA was first protected as the per-*O*-*t*-butyldimethylsilyl ether (per-*O*-TBS ether) **16** (**Figure 2**) (Kobayashi et al., 2013). A previously published X-ray crystal structure indicates that tetrabutylammonium fluoride (TBAF)-mediated deprotection of a per-*O*-silyl EA derivative occurs preferentially at the 3- and 3′-silyl ethers to afford the 3,3′-diphenolate *in situ*, presumably due to inductive effects of the proximal endocyclic lactone oxygen (Kobayashi et al., 2013).

The mild fluoride source TASF (Scheidt et al., 1998) effected removal of a single silyl ether *in situ* to generate the 3-monophenolate of EA, which underwent glycosylation in the presence of a per-*O*-acetyl α-glycopyranosyl iodide to furnish the desired 3-*O*-glycosides as single anomers. Glycosylation with α-L-rhamnopyranosyl iodide or α-D-xylopyranosyl iodide afforded the α-rhamnopyranoside **17** or β-xylopyranoside **18** of EA, respectively (**Figure 2**). Due to the *cis*-1,2 configuration of the α-xylosyl iodide donor, addition of tetrabutylammonium iodide (TBAI) was crucial to promote *in situ* anomerization of the sugar to the more reactive β-iodide (Gervay et al., 1997; Hadd and Gervay, 1999). This facilitated displacement of the equatorial iodide by the adjacent acetoxy substituent, providing solely the β-xyloside **18** upon reaction of the acetoxonium intermediate with the protected EA acceptor. The protected EAGs were then de-*O*-silylated using K_2_CO_3_ in wet DMF (Jiang and Wang, 2003) followed by de-*O*-acetylation using K_2_CO_3_ in MeOH/H_2_O. A final treatment with cation-exchange resin afforded the desired 3-*O*-glycosides of EA (**7**, **10**) (**Figure 2**).

The anomeric stereochemistry of the β-xylosides (**6**, **7**) was assigned based on the characteristic vicinal ^3^*J*_1,2_ coupling of approximately 10 Hz in the ^1^H NMR spectrum, indicative of diaxial coupling. However, the axial 2-hydroxy substituent of rhamnose and mannose precludes elucidation of anomeric stereochemistry based on ^3^*J*_1,2_ coupling, as both the α and β anomers have ^3^*J*_1,2_ coupling constants in the 0–3 Hz range (Pettit et al., 2011). Alternatively, the α configuration of phenolic rhamnosides (**8–10**) and mannosides (**4**, **5**) was deduced from the anomeric ^1^*J*_CH_ coupling constant of approximately 170 Hz, which is in accord with the typical magnitude for α-glycosides, compared to approximately 160 Hz for the corresponding β-glycosides (Pettit et al., 2011).

**FIGURE 3:**
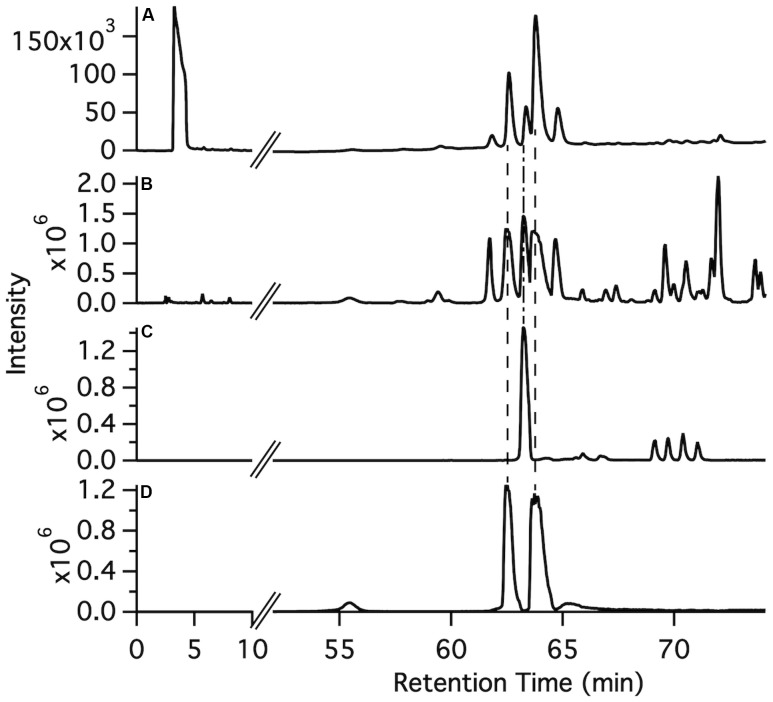
**Identification of ellagic acid glycosides in the 220D-F2 extract. (A)** Total UV-visible absorbance (210–500 nm) chromatogram. **(B)** FTMS base peak chromatogram. **(C)** FTMS chromatogram filter for ellagic acid rhamnoside *m/z* range (447–448). **(D)** FTMS chromatogram filter for ellagic acid xyloside *m/z* range (433–434). Comparison of fragmentation patterns from compounds identified in 220D-F2 extract and synthetic standards can be found in Supplementary Figure S1.

The synthetic compounds were used as standards for liquid chromatography–mass spectrometry (LC–MS) to probe for the occurrence of the various glycosylated aromatic compounds in the 220D-F2 extract. Both EA xyloside (**7**) and EA rhamnoside (**10**) were identified in the complex extract (**Figure 3**). However, the glycosylated phenols and catechols were not observed (Supplementary Figures S1–S7).

The panel of compounds was assayed to determine the effects on *S. aureus* growth and biofilm formation (**Table 1** and Supplementary Figures S8, S9). The 220D-F2 extract and EA also were included as controls (Quave et al., 2012). Among the compounds tested, phenol (**2**), EA xyloside (**7**), phenol rhamnoside (**8**), catechol rhamnoside (**9**), and EA rhamnoside (**10**) inhibited biofilm formation. However, EA xyloside exhibited a lower MIC_50_ (32 μg/mL) than MBIC_50_ (64 μg/mL). In addition, only EA rhamnoside (**10**) was able to inhibit 90% of biofilm formation. Furthermore, CLSM of *S. aureus* biofilms treated with EA rhamnoside (**10**) showed decreased biofilm formation, with decreased tower heights and attachment, as compared to controls.

**Table 1:** Minimum concentrations of extract and synthetic compounds required to inhibit biofilm formation (MBIC) or growth (MIC) of *S. aureus*.

Compound	MBIC_50_ (μg/mL)	MBIC_90_ (μg/mL)	MIC_50_ (μg/mL)	MIC_90_ (μg/mL)
220D-F2	25	100	512	ND
Ellagic acid (**1**)	128	ND	ND	ND
Phenol (**2**)	64	ND	ND	ND
Catechol (**3**)	ND	ND	ND	ND
Phenol mannoside (**4**)	ND	ND	512	ND
Catechol mannoside (**5**)	ND	ND	ND	ND
Phenol xyloside (**6**)	ND	ND	ND	ND
EA xyloside (**7**)	64	ND	32	ND
Phenol rhamnoside (**8**)	64	ND	ND	ND
Catechol rhamnoside (**9**)	128	ND	512	ND
EA rhamnoside (**10**)	64	128	ND	ND

*ND, not detected; >512 μg/mL*.

Assessment of combinations of single compounds identified in the extract 220D-F2 (EA, EA rhamnoside, and EA xyloside) by two-dimensional checkerboard assays resulted in lower MBIC_50_ values for all three. However, the combinations could not be confirmed as being strongly synergistic as all values were above the ΣFIC synergy cutoff of 0.5, and yet below the cutoff for additive effects (ΣFIC of 1) (**Table 2**). These data suggest that the increased efficacy of extract 220D-F2 may result from synergy between EAGs and other unidentified compounds in the extract.

**Table 2:** Fractional inhibitory concentration indices of biofilm inhibition for combinations of compounds identified in extract 220D-F2.

Compound combination	Lowest concentration with ≤50% biofilm formation (μg/mL)			ΣFIC
	Ellagic acid	EA rhamnoside	EA xyloside	
EA + EA rhamnoside	64	8	–	0.625
EA + EA xyloside	64	–	16	0.750
EA rhamnoside + EA xyloside	–	32	16	0.750

## Discussion

As phenol and catechol are key structural components of EA (**Figures 1**, **2**) and other phenolic compounds have shown anti-biofilm and anti-quorum sensing activity (Cowan, 1999; Brielmann, 2010; Gutierrez-Barranquero et al., 2015; Onsare and Arora, 2015; Zhou et al., 2015; Tsou et al., 2016), they were chosen as targets to determine the structural elements of the EA core required for bioactivity. Phenol and catechol containing compounds are widely observed in plant natural products (Cowan, 1999; Brielmann, 2010), suggesting that they may occur within *R. ulmifolius*. In addition, inclusion of phenol and catechol in the panel allowed for interrogation of the role of redox chemistry in EA anti-biofilm activity, as the reduction potential of EA lies between the more reducing catechol and the less reducing phenol (Steenken and Neta, 1982). The selection of rhamnose and xylose for attachment to the core structures was made based on the molecular weights of the previously identified EAGs (Quave et al., 2012; Talekar et al., 2014) and the ubiquity of rhamnose and xylose in plants (Cowan, 1999). Mannose was included to determine if general changes that occur upon sugar attachment (solubility, hydrogen bonding, etc.) were sufficient to elicit activity.

The panel of compounds was synthesized by coupling the appropriate aromatic core to per-*O*-acetyl glycoside (Donahue and Johnston, 2006; Camponovo et al., 2009; Yu and Kizhakkedathu, 2010) or per-*O*-acetyl α-glycosyl iodide donors (Gervay and Hadd, 1997; Gervay et al., 1997; Hadd and Gervay, 1999; Bhat and Gervay-Hague, 2001; Du and Gervay-Hague, 2005; Schombs et al., 2010) (for phenol/catechol and EA, respectively). Given the insolubility and low reactivity of EA, EA was protected as the per-*O*-TBS ether and then regioselectively deprotected using TASF, which afforded the singly glycosylated products EA xyloside (**7**) and EA rhamnoside (**10**).

Previous work demonstrated that *R. ulmifolius* extracts inhibit biofilm formation of *S. aureus* at lower concentrations than required for inhibition of growth (Quave et al., 2012). Therefore, the minimum inhibitory concentrations necessary for inhibition of 50 and 90% growth (MIC_50_ and MIC_90_) were tested for all compounds, in addition to testing anti-biofilm activity. These studies determined whether the observed anti-biofilm activity is influenced by growth inhibitory activity of the compounds, or if the effects were solely based on inhibiting biofilm formation. A clinical osteomyelitis strain (UAMS-1) strain was used for all assays and its biofilm deficient mutant (UAMS-929; Δ*sarA*) was used as a control. Selection of this strain was based on its ability to reproducibly create robust biofilms, its direct relevance to the clinic, and widespread use in *S. aureus* pathogenesis studies. In addition to synthetic EAGs, the previously identified anti-biofilm extract (220D-F2), EA, and core phenolic compounds also were tested for activity to evaluate the structure–activity relationship.

From the anti-biofilm studies, the identity of the sugar was identified as a key determinant of biological activity. Neither mannose nor xylose derivatives of phenol (**4**, **6**) and catechol (**5**) showed anti-biofilm or anti-microbial activity; however, the rhamnose derivatives (**8**, **9**) did exhibit potent anti-biofilm activity. As mannose was not identified as a potential sugar in the MS characterization of 220D-F2, these results suggest that plant-derived sugars exert differential effects on microbial biofilm formation. In addition, the catechol moiety, and therefore potential redox activity, is not required for anti-biofilm activity (**Table 1**), as phenol/phenol glycosides (**2**, **8**) are able to inhibit biofilm formation more potently than catechol/catechol glycosides (**3**, **9**).

Of the synthetic compounds tested, only rhamnose derivatives were able to inhibit biofilm formation at concentrations lower than the growth inhibitory concentration. Rhamnose derivatives of phenol (**8**), catechol (**9**), and EA (**10**) all inhibited biofilm formation at lower concentrations than their parent phenolic compounds and had even lower growth inhibition than the 220D-F2 extract. In contrast, the xylose derivative of phenol (**6**) did not show any measurable activity, while EA xyloside (**7**) inhibited growth at half the concentration of biofilm inhibition (Supplementary Figures S8, S9). These data demonstrate that the phenolic core and the attached sugar both play important roles in toxicity and anti-biofilm activity. Furthermore, this work suggests that the appended sugars are not simply modulating compound solubility, and therefore the ability to reach the cellular target(s), but instead indicate that the sugars may be making key interactions with cellular components, thereby increasing anti-biofilm activity.

To determine if the synthetic compounds are components of the 220D-F2 extract, LC–MS/MS analysis of 220D-F2 and all of the synthetic compounds was performed (**Figure 3** and Supplementary Figures S1–S7). Many of the compounds elute as multiple peaks in the LC trace, likely due to formation of non-covalent dimers (as observed by MS) that can access multiple relative orientations. As can be seen in **Figure 3**, EA xyloside (**7**) and EA rhamnoside (**10**) were identified in the 220D-F2 extract based on retention time and fragmentation patterns that matched to authentic standards (**Figure 3** and Supplementary Figures S1, S3, S4). These data show that EAGs not only exhibit anti-biofilm activity, but also are active components that contribute to anti-biofilm activity of the previously identified 220D-F2 extract, highlighting the roles of EAGs in 220D-F2 bioactivity.

**FIGURE 4:**
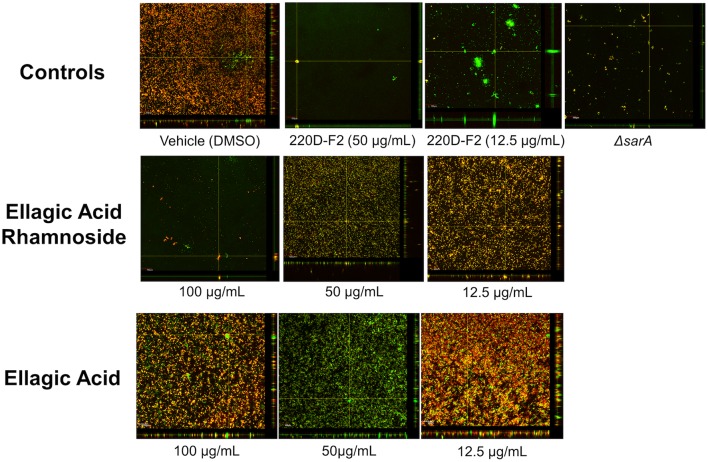
**Impact on biofilm formation as assessed by confocal laser scanning microscopy**. Biofilm inhibition assays were undertaken with UAMS-1 and its isogenic sarA mutant (UAMS-929) as a control. An orthogonal view is included to illustrate overall biofilm architecture at a magnification of 10×.

The high anti-biofilm activity of EA rhamnoside, coupled with limited growth inhibition (Supplementary Figure S8), suggests that it is a key component contributing to anti-biofilm activity of the 220D-F2 extract. To further investigate how biofilm formation is inhibited, CLSM was used to assess biofilm structure. Treatment with sub-inhibitory levels of 220D-F2 previously has been shown to disrupt biofilm structure and lead to formation of tower-like structures.(Quave et al., 2012) Concentrations of 12.5 μg/mL of 220D-F2 yielded limited attachment of biofilm and max tower heights of 116 μm (**Figure 4**). At 50 μg/mL, less attachment was evident and max tower heights of 48 μm were observed. Similarly to 220D-F2, 100 μg/mL of EA rhamnoside demonstrated max tower height of 44 μm and limited attachment, in line with what was observed in the *sarA* mutant control.

## Conclusion

EA rhamnoside has been identified as an inhibitor of biofilm formation, without concomitant inhibition of bacterial growth. Glycosylation of phenolic compounds significantly modulates both biofilm and growth inhibitory activity, suggesting that there may be key interactions between the sugar and biological targets. EA rhamnosides and xyloside have been identified within the 220D-F2 extract, highlighting their roles in the previously observed anti-biofilm activity. Furthermore, identification of single compounds with anti-biofilm activity will allow for future studies to elucidate the mechanism of biofilm inhibition.

## Author Contributions

BF, KN, JL, CQ, and EW designed the study. BF, KN, JL, PJ, and JG-R performed all experiments. BF, KN, JL, CQ, and EW analyzed the data. BF, KN, JL, CQ, and EW wrote the manuscript.

## Conflict of Interest Statement

The authors declare that the research was conducted in the absence of any commercial or financial relationships that could be construed as a potential conflict of interest.
