# Supplementary material for: Identification of Ellagic Acid Rhamnoside as a Bioactive Component of a Complex Botanical Extract with Anti-biofilm Activity
Source: Front Microbiol. 2017 Mar 23;8:496. doi: 10.3389/fmicb.2017.00496 (PMC5362615; doi:10.3389/fmicb.2017.00496)
Supplement: Supplementary file 1 [file Data_Sheet_1.pdf]

Supplementary Material

Identification of Ellagic Acid Rhamnoside as a Bioactive Component of a Complex Botanical Extract with Anti-biofilm Activity

Benjamin M. Fontaine[ξ 1], Kate Nelson[ξ2*], James T. Lyles[3], Parth P. Jariwala [1,3], Jennifer M. García-Rodriguez[1], Cassandra L. Quave[2,3]*, Emily E. Weinert[1]*

*** Correspondence:** Cassandra L. Quave: cassandra.leah.quave@emory.edu; Emily E. Weinert: emily.weinert@emory.edu

1 Supplementary Data

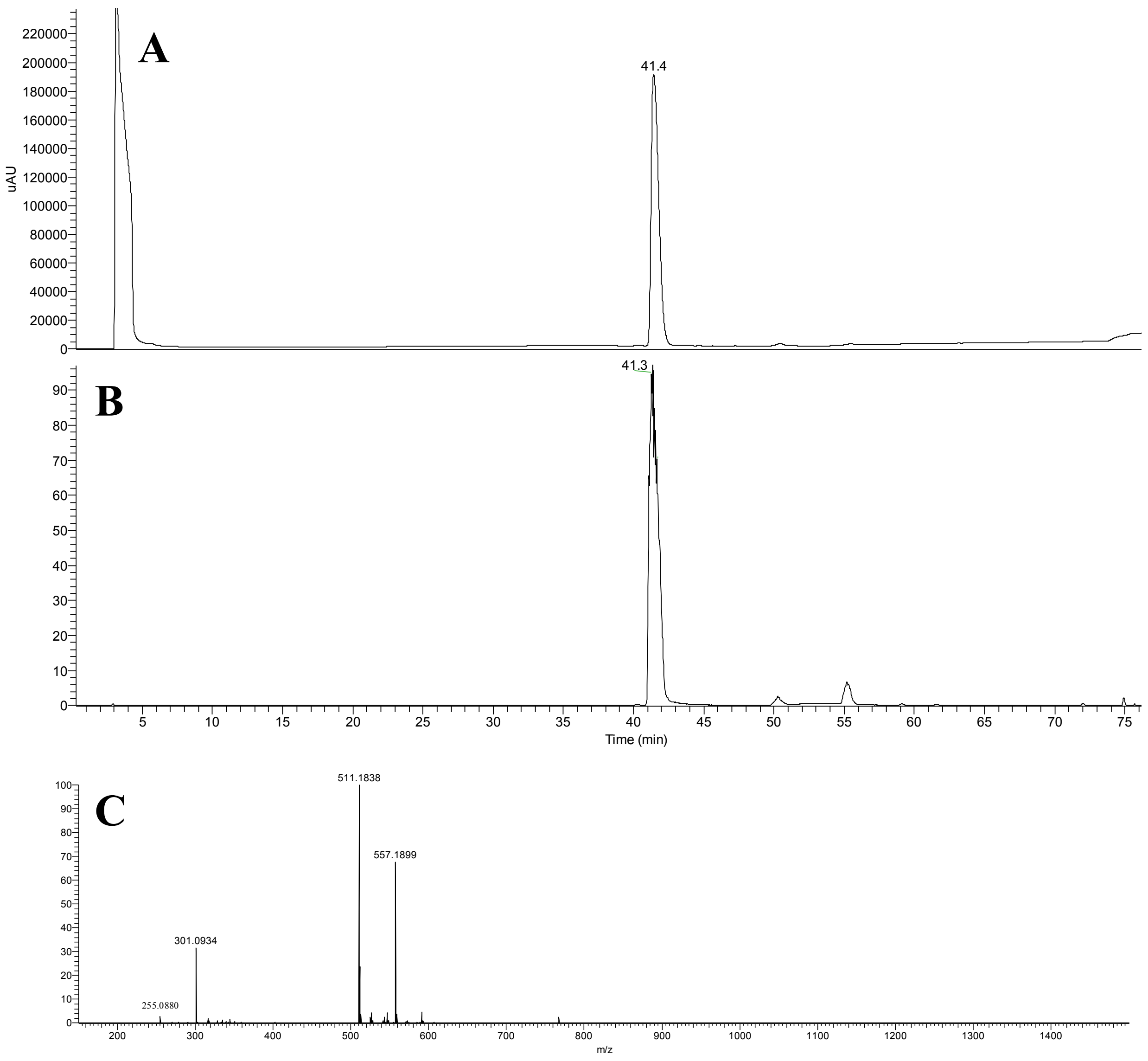

Figure S1: FT-MS ESI negative spectra of (**A**) ellagic acid rhamnoside eluting at 63.3 min, (**B**) ellagic acid xyloside eluting at 62.5 min, and (**C**) ellagic acid xyloside 63.7 min in extract 220D-F2.

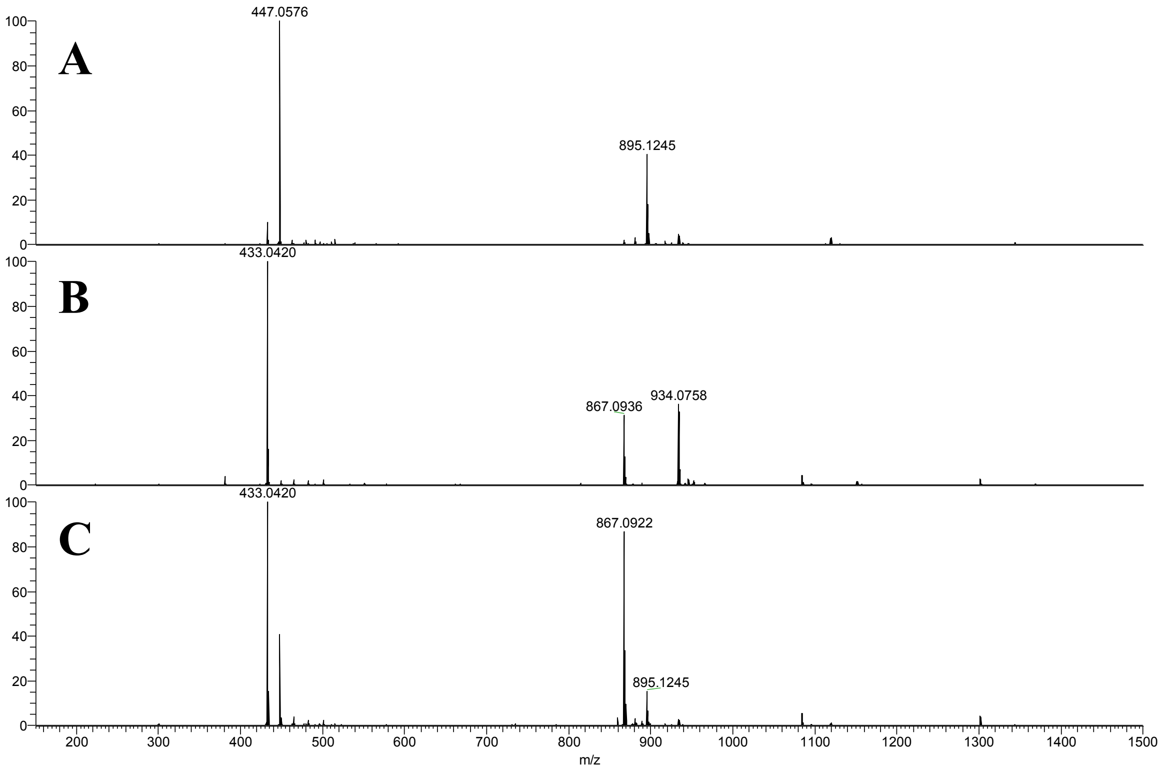

Figure S2: Catechol rhamnoside (**A**) UV-Vis chromatogram 210-500 nm total spectrum, (**B**) base peak FT-MS chromatogram, and (**C**) mass spectra of peak at 41.3 min showing $[M\text{-}H]^-$ at *m/z* 255.0880, $[M\text{+formate-}H]^-$, $[2M\text{-}H]^-$, and $[2M\text{+formate-}H]^-$ ions.

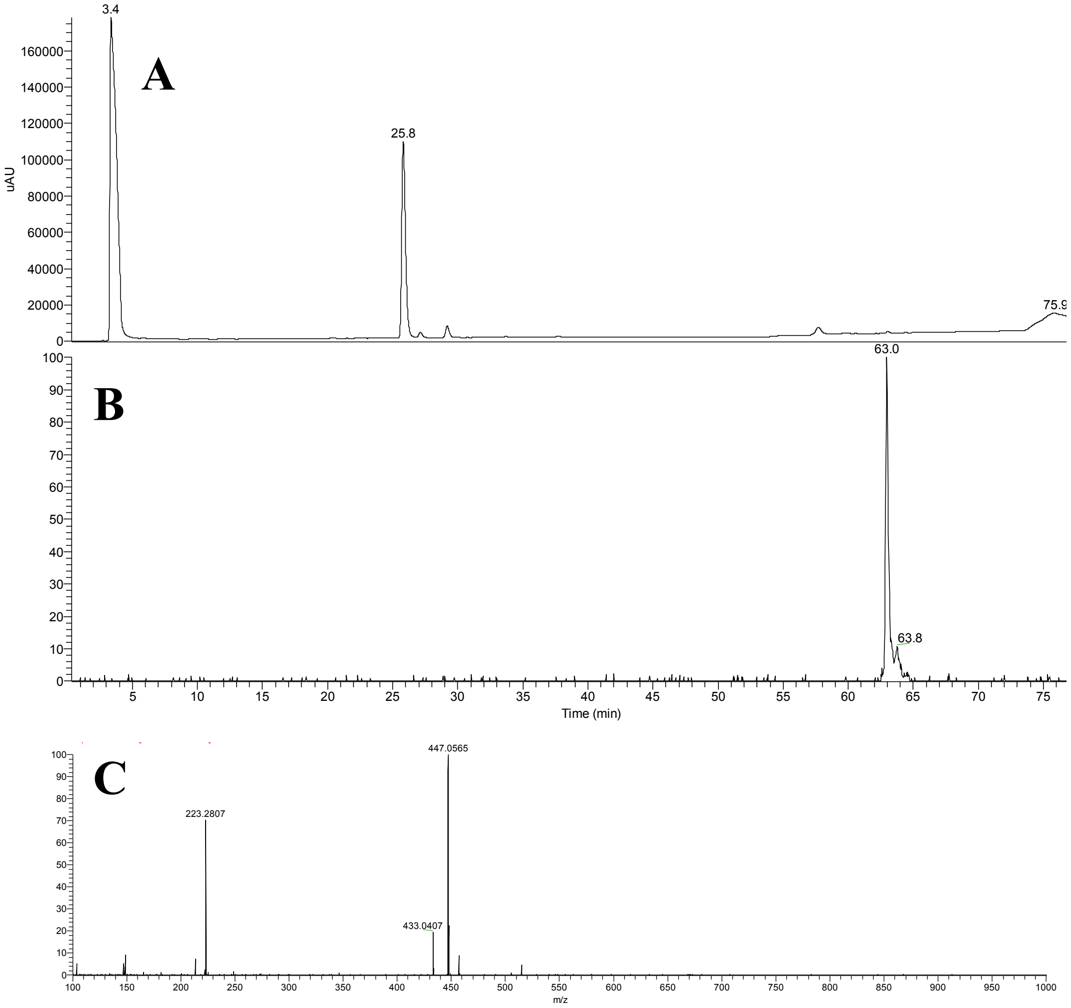

Figure S3: Ellagic acid rhamnoside (**A**) UV-Vis chromatogram 210-500 nm total spectrum, (**B**) FT-MS chromatogram showing *m/z* 447-448, and mass spectra.

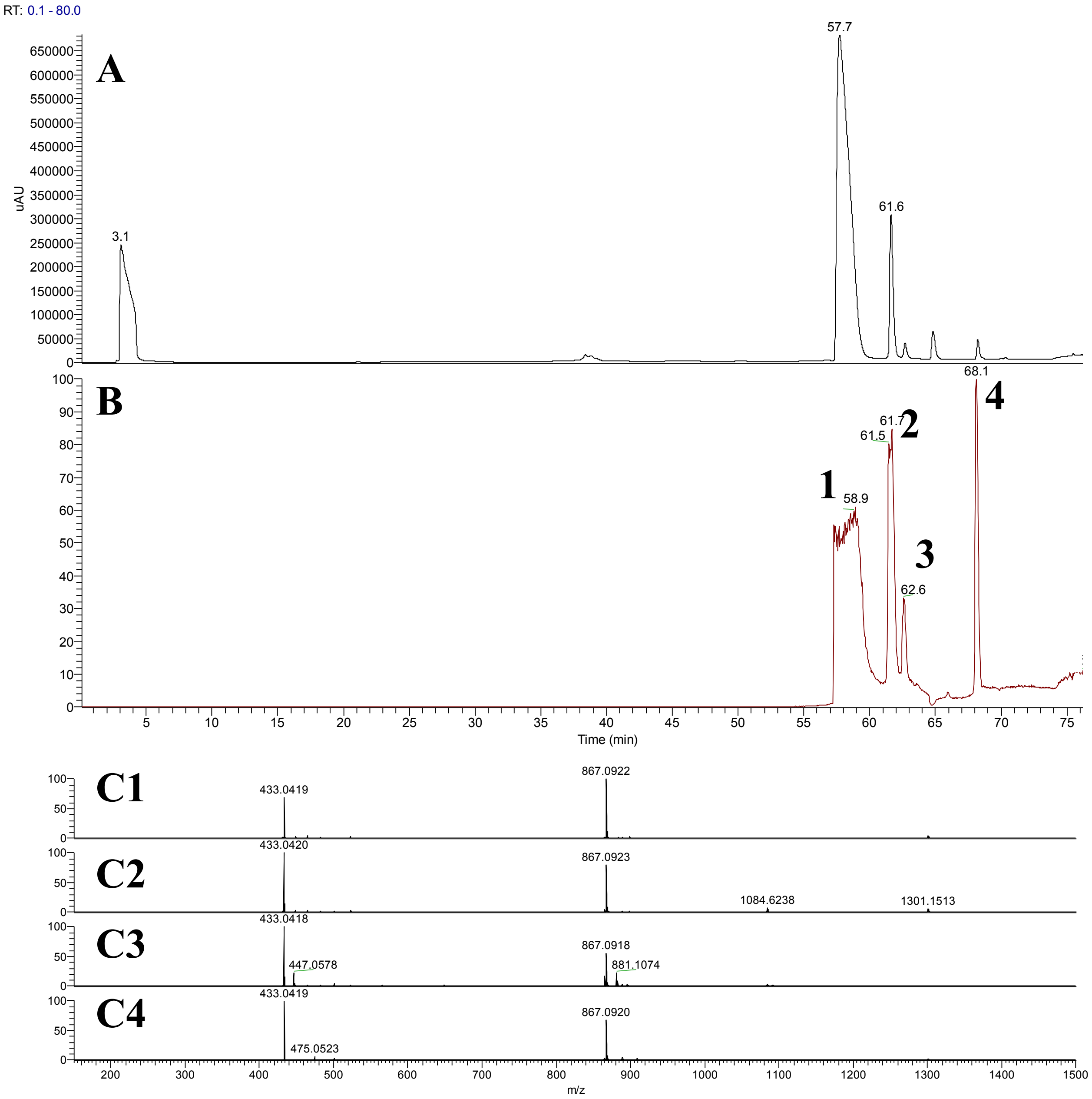

Figure S4: Ellagic acid xyloside (**A**) UV-Vis chromatogram, (**B**) FT-MS chromatogram showing *m/z* 433-434, and (**C**) mass spectra of peaks **1**, **2**, **3**, **4**.

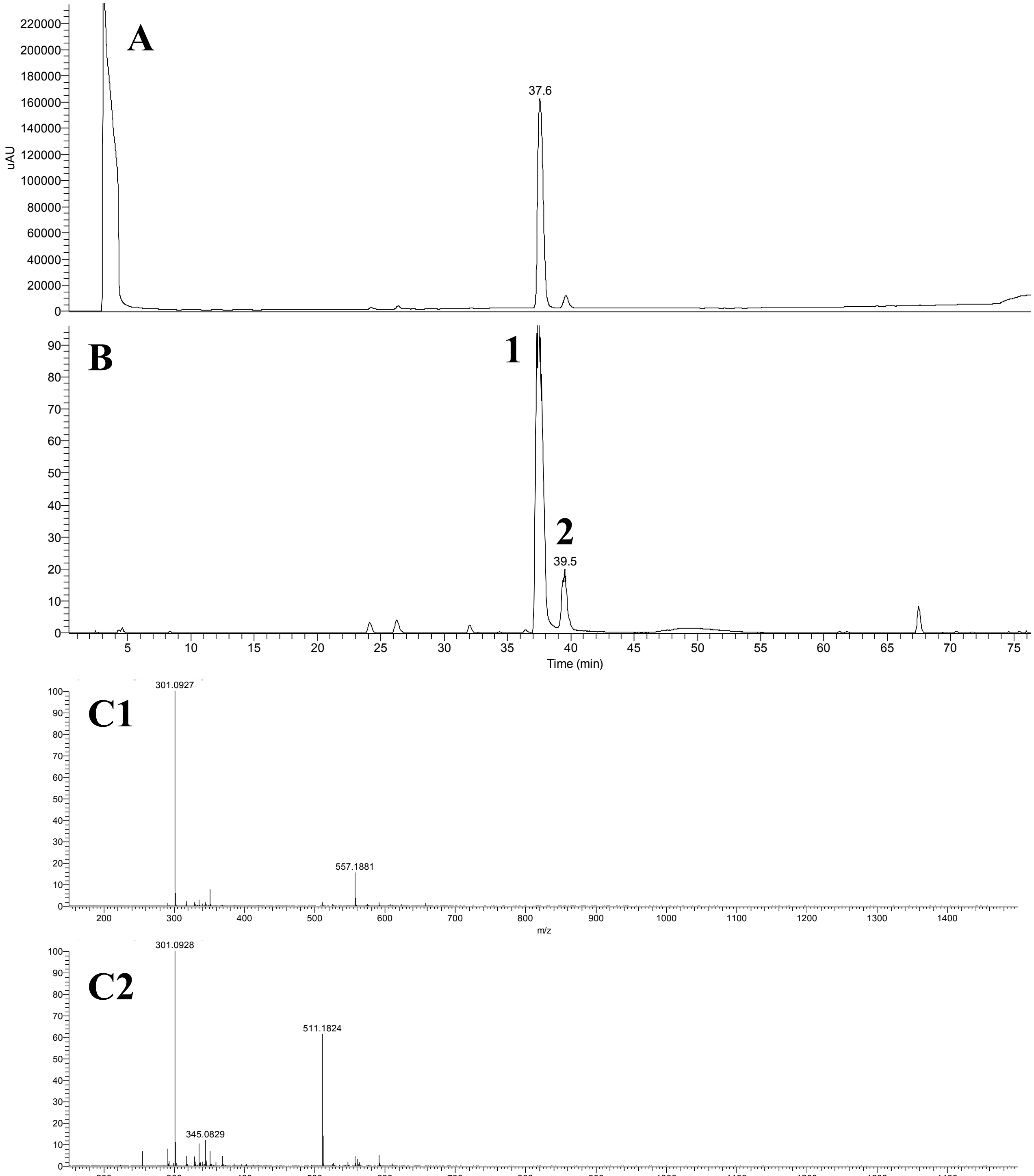

Figure S5: Phenyl glycoside (**A**) UV-Vis chromatogram 210-500 nm total spectrum, (**B**) base peak FT-MS chromatogram, (**C1**) mass spectra of peak at 37.3 min showing [M+formate-H]$^-$ and [2M+formate-H]$^-$ ions at *m/z* 301.0927and 557.1881, respectively, and (**C2**) mass spectra of peak at 39.5 min showing [M+formate-H]$^-$ at *m/z* 301.0928 and [2M-H]$^-$ at *m/z* 511.1824.

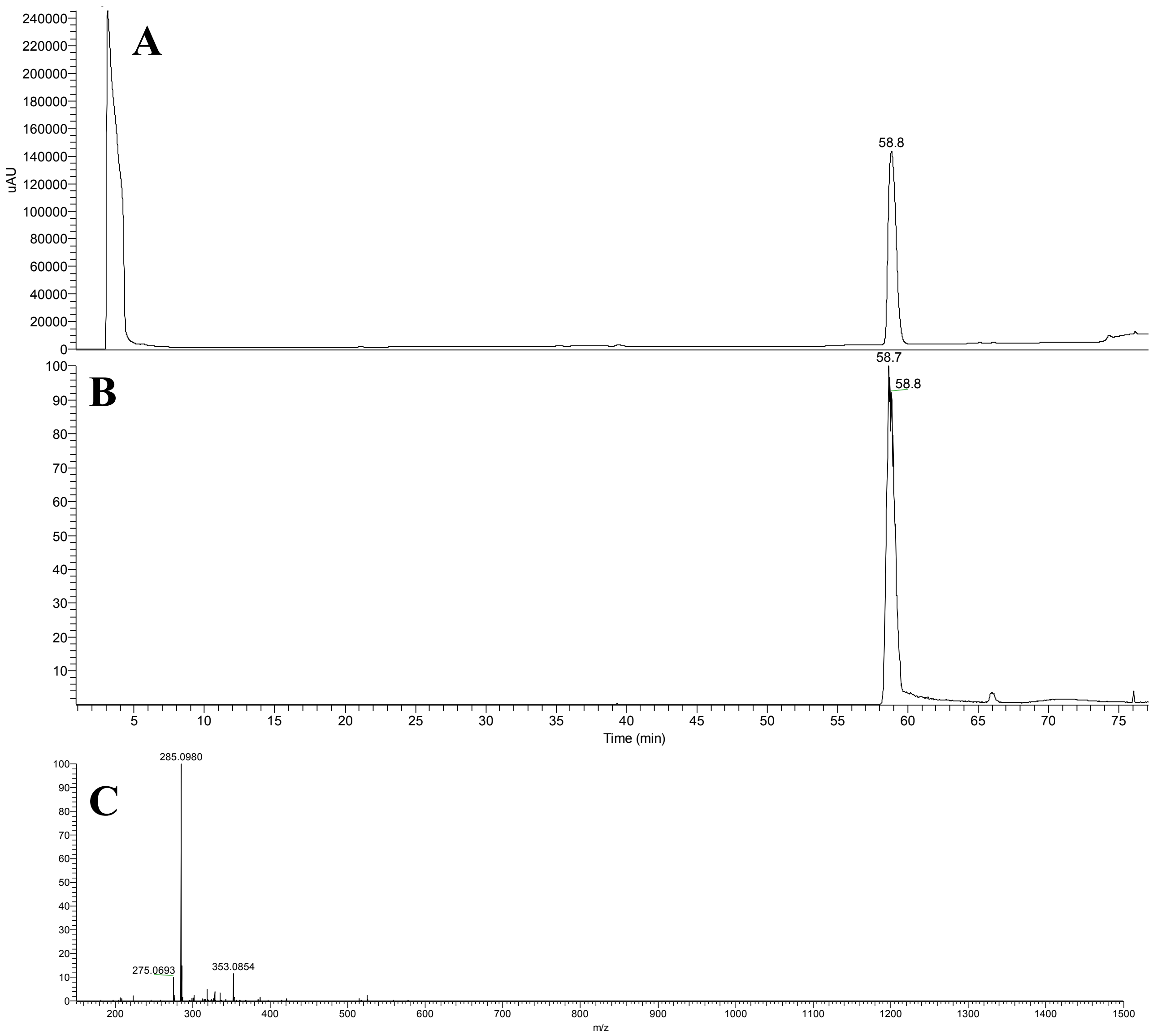

Figure S6: Phenyl rhamnoside (**A**) UV-Vis chromatogram 210-500 nm total spectrum, (**B**) base peak FT-MS chromatogram, and (**C**) mass spectra of peak at 58.7 min showing, $[M+formate-H]^-$at *m/z* 285.0980.

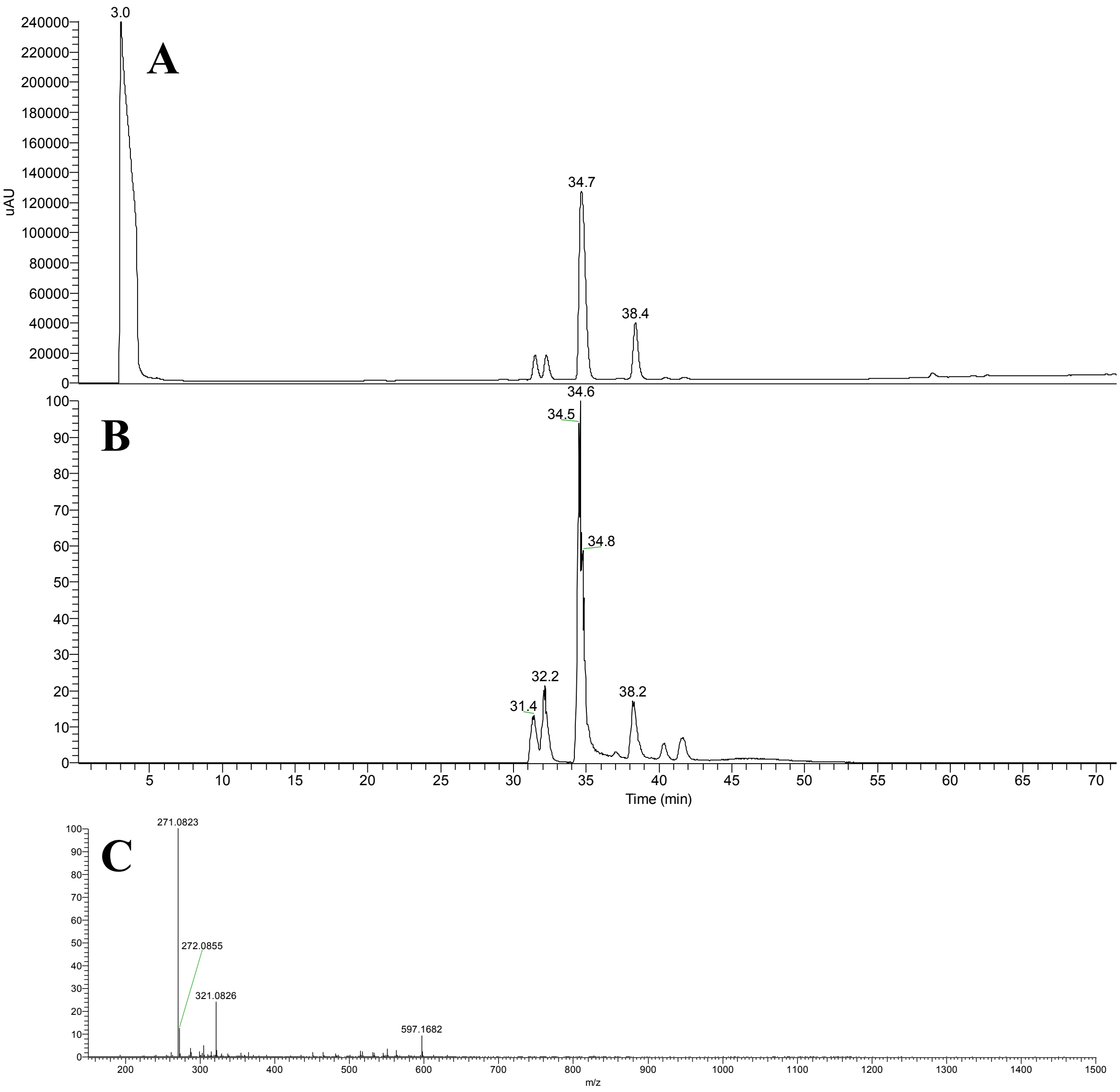

Figure S7: Phenyl xyloside (**A**) UV-Vis chromatogram 210-500 nm total spectrum, (**B**) base peak FT-MS chromatogram, and (**C**) mass spectra of peak at 34.6 min showing, $[M+formate-H]^-$at *m/z* 271.0823.

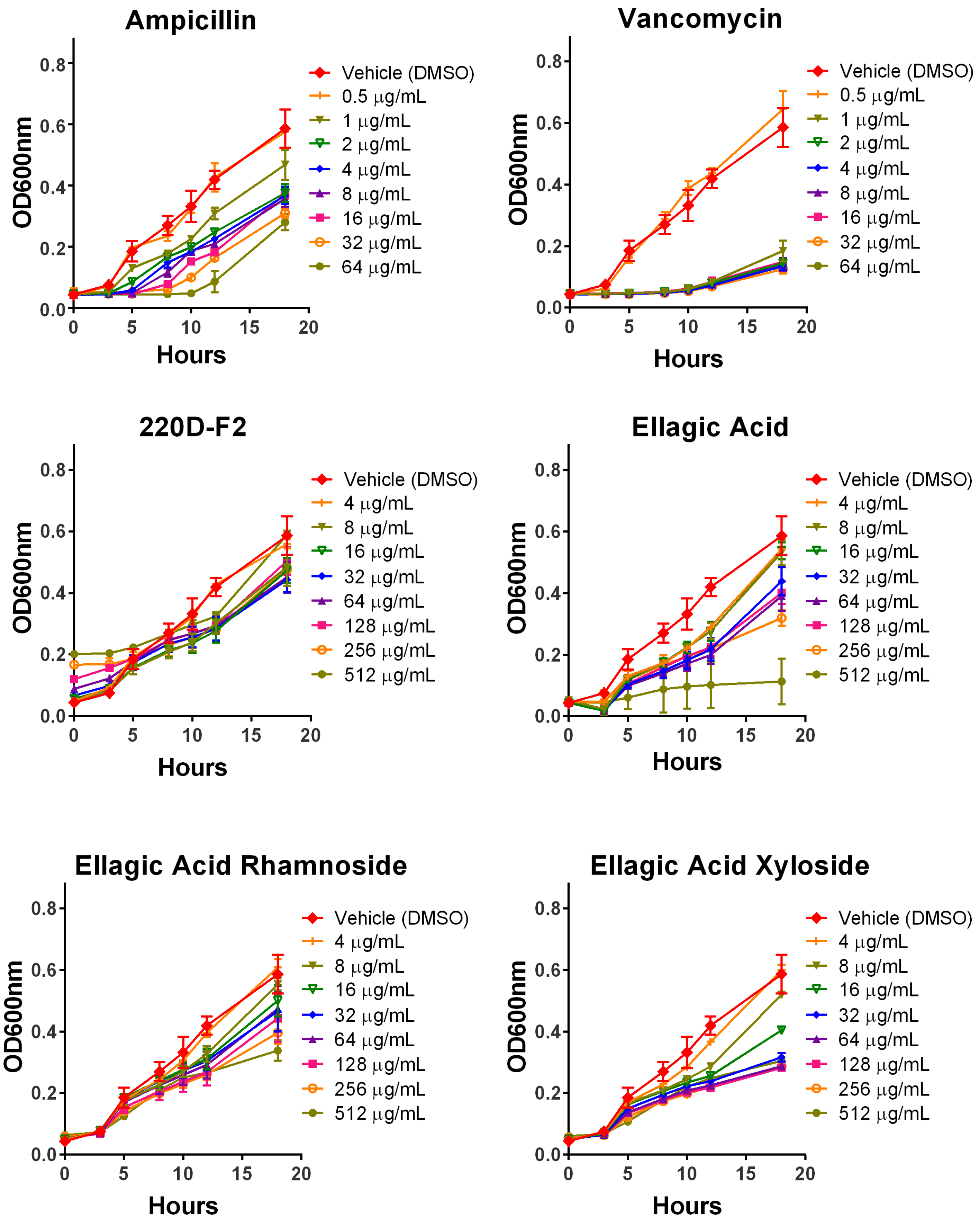
Ampicillin
OD600nm
Hours
0.0
0.2
0.4
0.6
0.8
0
5
10
15
20
Vehicle (DMSO)
0.5 µg/mL
1 µg/mL
2 µg/mL
4 µg/mL
8 µg/mL
16 µg/mL
32 µg/mL
64 µg/mL
Vancomycin
OD600nm
Hours
Vehicle (DMSO)
0.5 µg/mL
1 µg/mL
2 µg/mL
4 µg/mL
8 µg/mL
16 µg/mL
32 µg/mL
64 µg/mL
220D-F2
OD600nm
Hours
Vehicle (DMSO)
4 µg/mL
8 µg/mL
16 µg/mL
32 µg/mL
64 µg/mL
128 µg/mL
256 µg/mL
512 µg/mL
Ellagic Acid
OD600nm
Hours
Vehicle (DMSO)
4 µg/mL
8 µg/mL
16 µg/mL
32 µg/mL
64 µg/mL
128 µg/mL
256 µg/mL
512 µg/mL
Ellagic Acid Rhamnoside
OD600nm
Hours
Vehicle (DMSO)
4 µg/mL
8 µg/mL
16 µg/mL
32 µg/mL
64 µg/mL
128 µg/mL
256 µg/mL
512 µg/mL
Ellagic Acid Xyloside
OD600nm
Hours
Vehicle (DMSO)
4 µg/mL
8 µg/mL
16 µg/mL
32 µg/mL
64 µg/mL
128 µg/mL
256 µg/mL
512 µg/mL

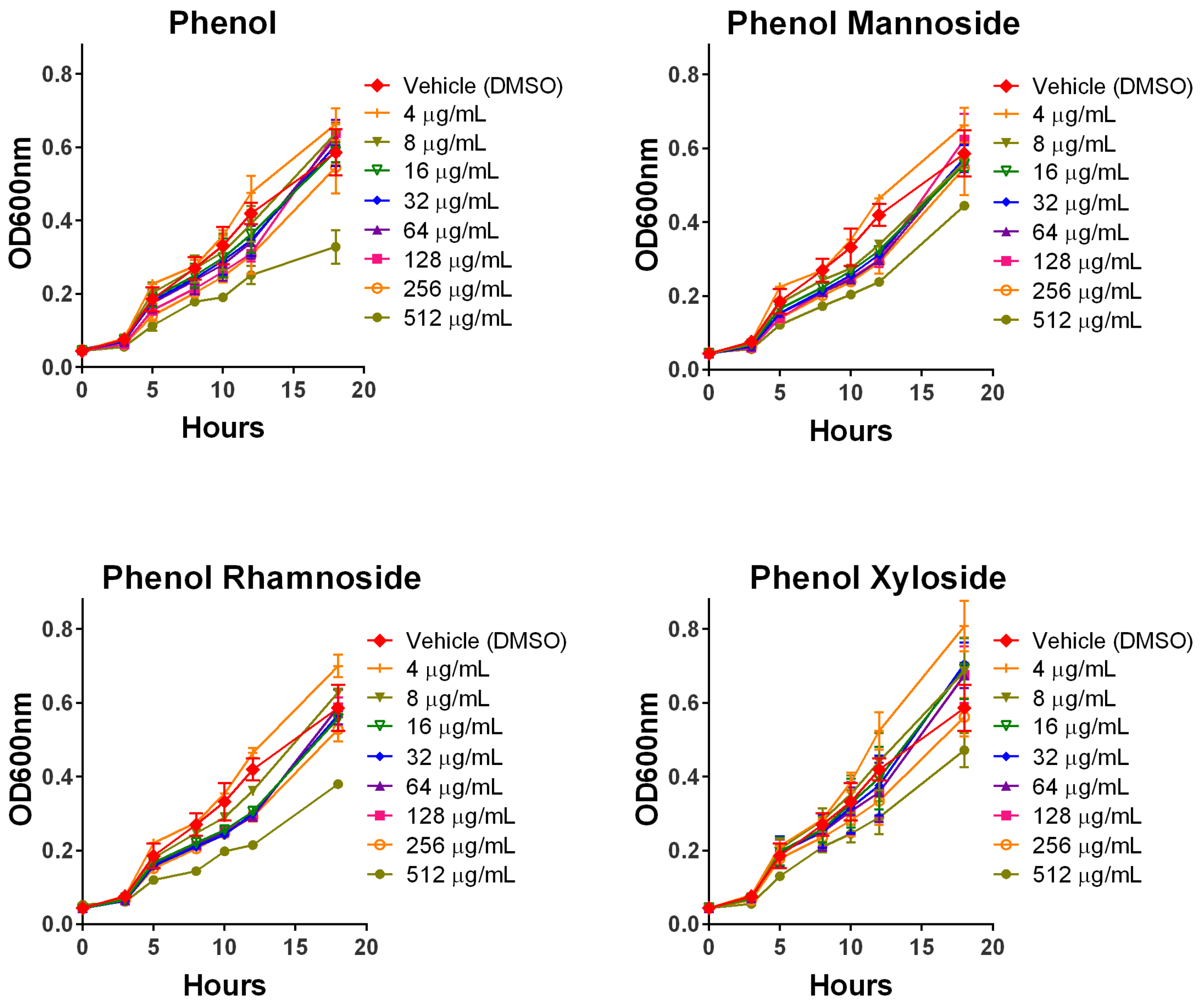
Phenol
Phenol Mannoside
Phenol Rhamnoside
Phenol Xyloside
OD600nm
Hours
0.0
0.2
0.4
0.6
0.8
0
5
10
15
20
Vehicle (DMSO)
4 µg/mL
8 µg/mL
16 µg/mL
32 µg/mL
64 µg/mL
128 µg/mL
256 µg/mL
512 µg/mL

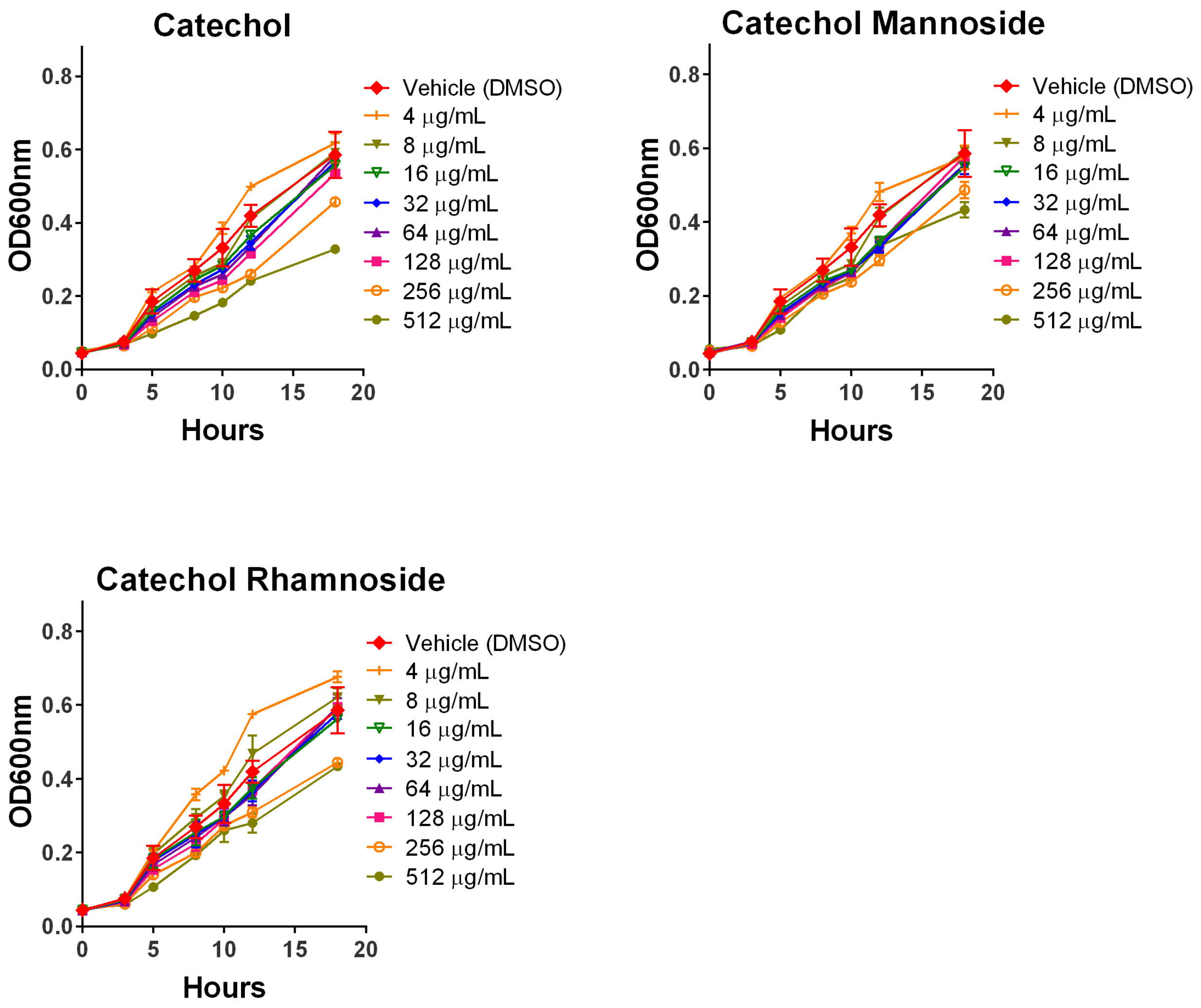

Figure S8: Growth curves for controls, 220D-F2 and synthesized analogs.

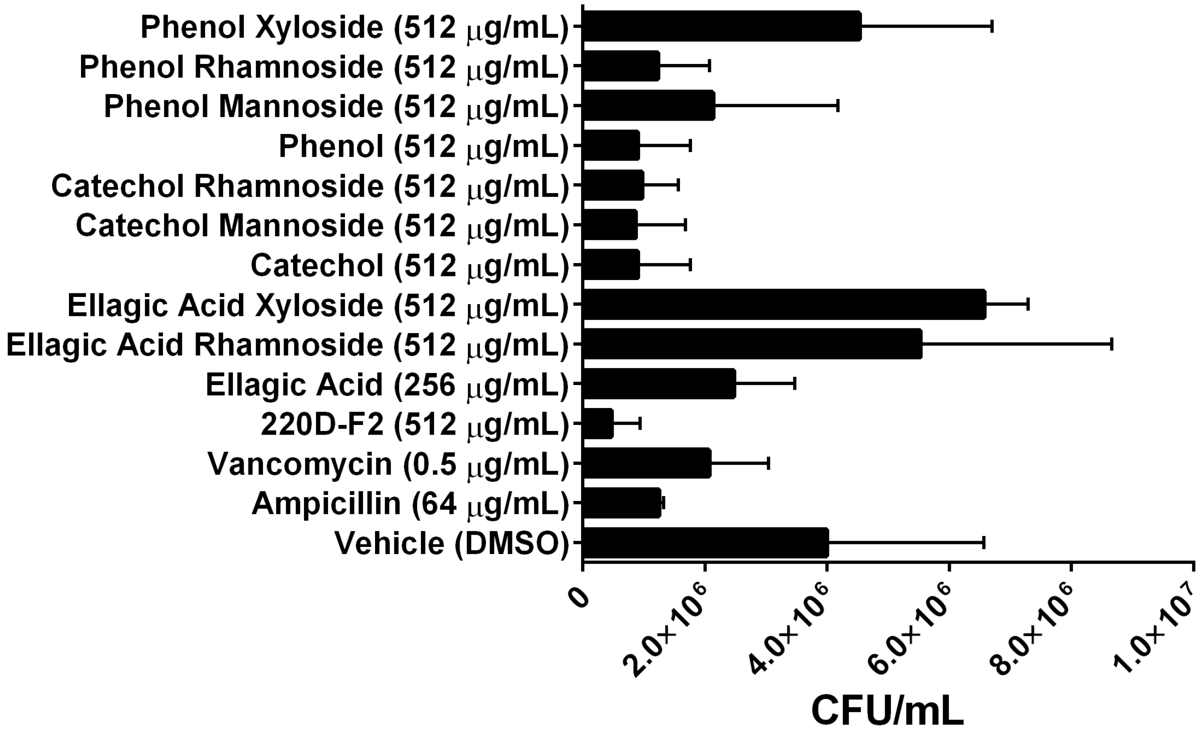

Figure S9: Total CFU/mL following 18 h incubation. None of the above represented concentrations exhibited a statistically significant (P<0.05) difference from the vehicle (DMSO) control.

NMR Spectra

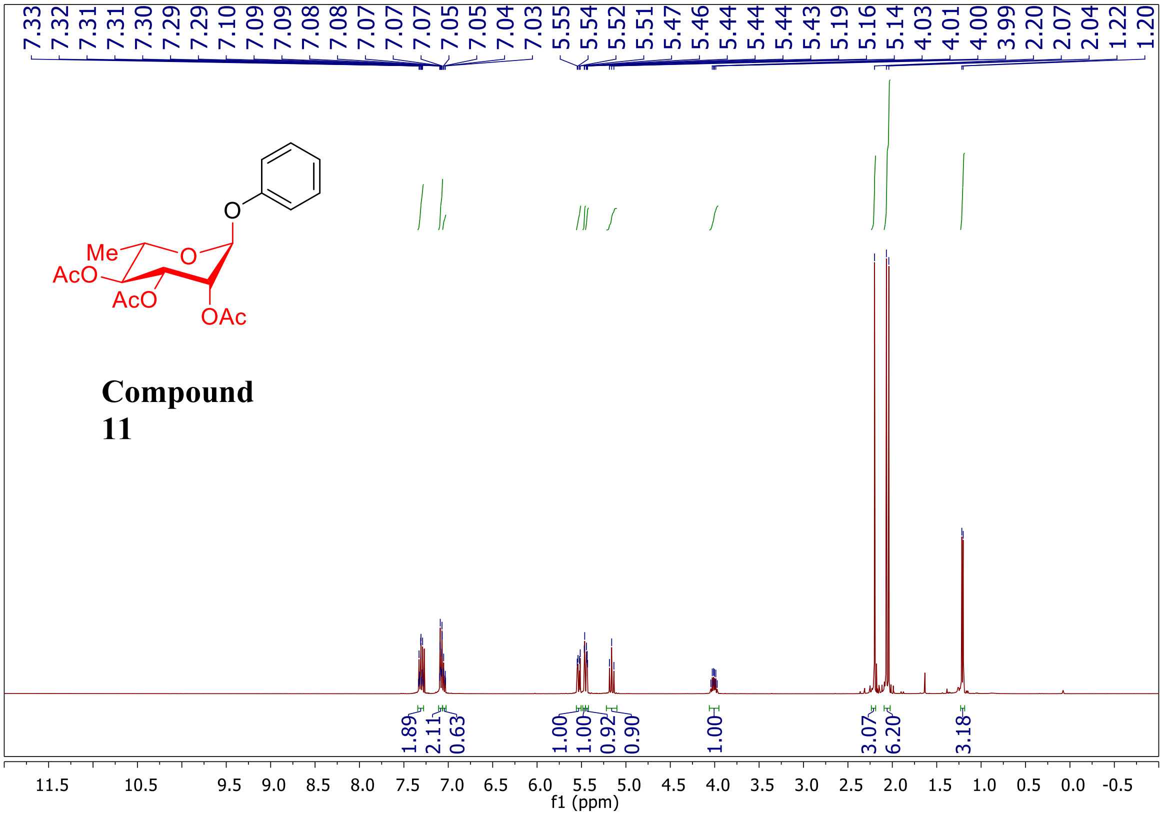

Compound 11

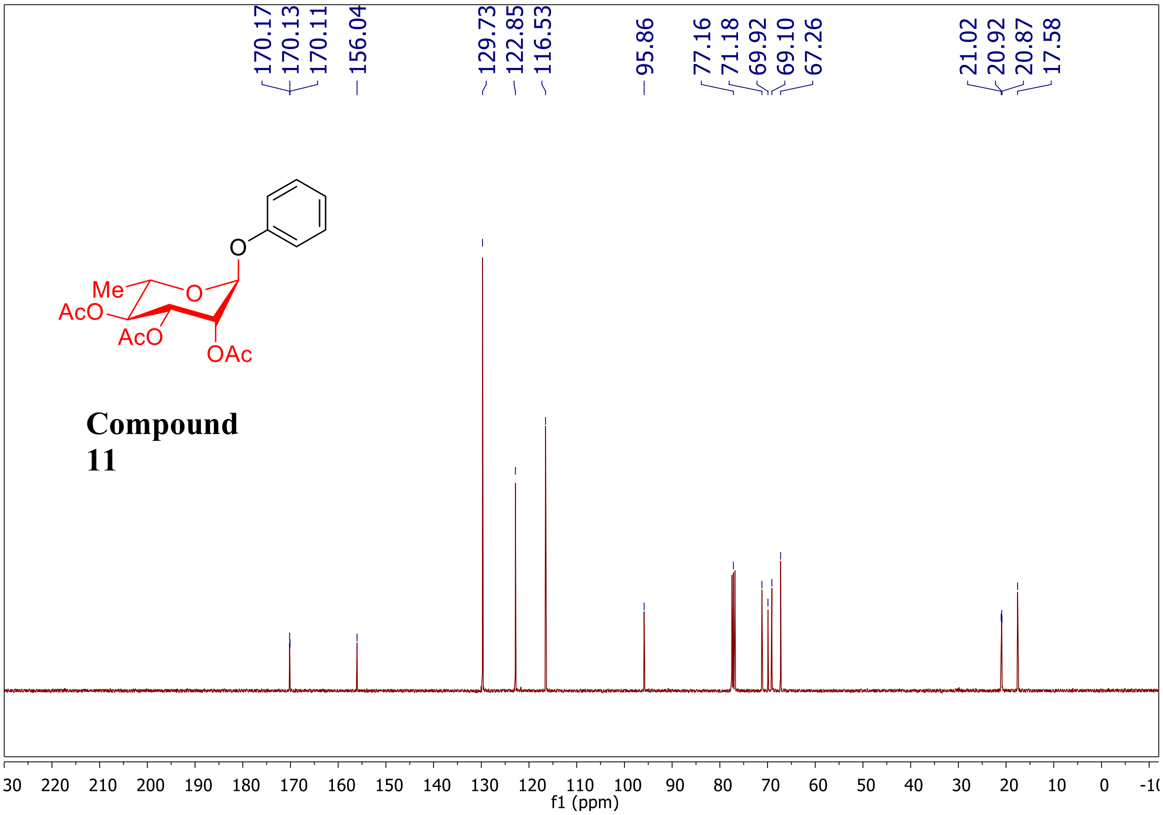
170.17
170.13
170.11
156.04
129.73
122.85
116.53
95.86
77.16
71.18
69.92
69.10
67.26
21.02
20.92
20.87
17.58
Me
O
AcO
AcO
OAc
Compound 11
f1 (ppm)

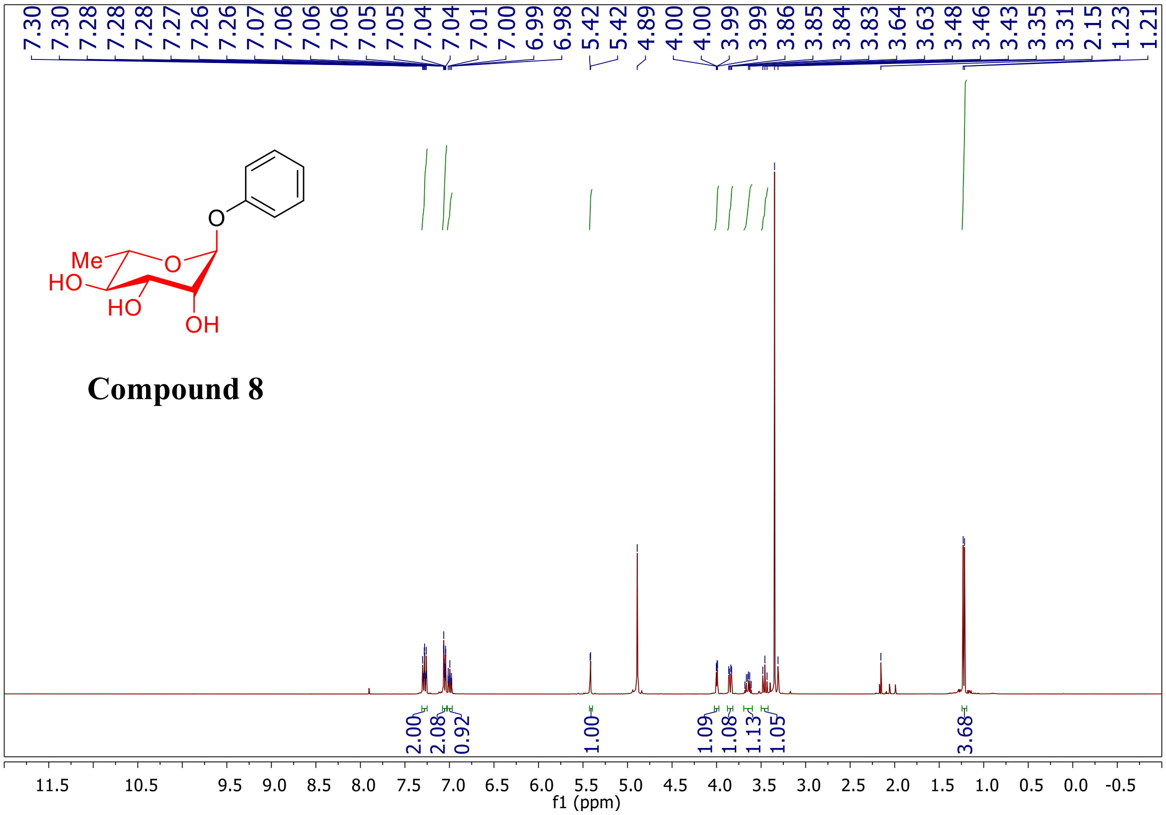
7.30
7.30
7.28
7.28
7.28
7.27
7.26
7.26
7.07
7.06
7.06
7.06
7.05
7.05
7.04
7.04
7.01
7.00
6.99
6.98
5.42
5.42
4.89
4.00
4.00
3.99
3.99
3.86
3.85
3.84
3.83
3.64
3.63
3.48
3.46
3.43
3.35
3.31
2.15
1.23
1.21
Me
O
HO
HO
OH
Compound 8
2.00
2.08
0.92
1.00
1.09
1.08
1.13
1.05
3.68
f1 (ppm)

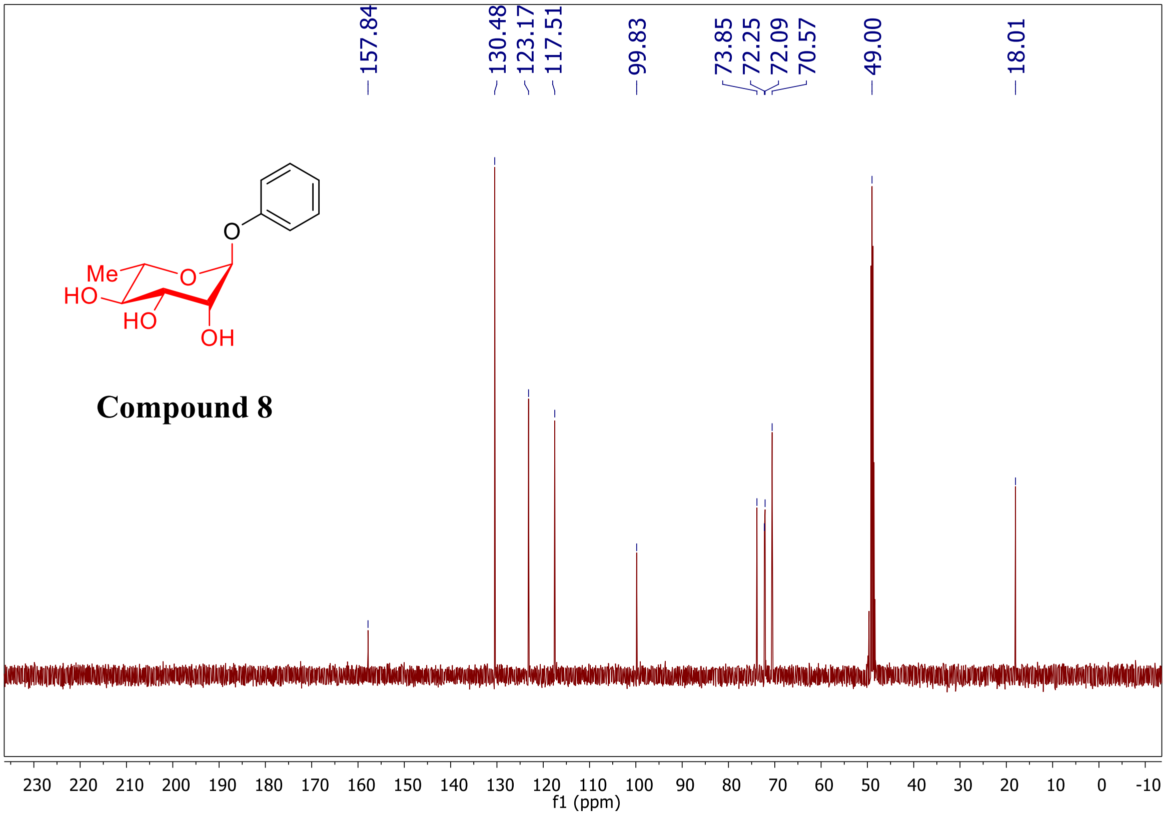

^{13}C-^{1}H coupled NMR (anomeric $^{1}J_{CH}$ = 169.96 Hz)

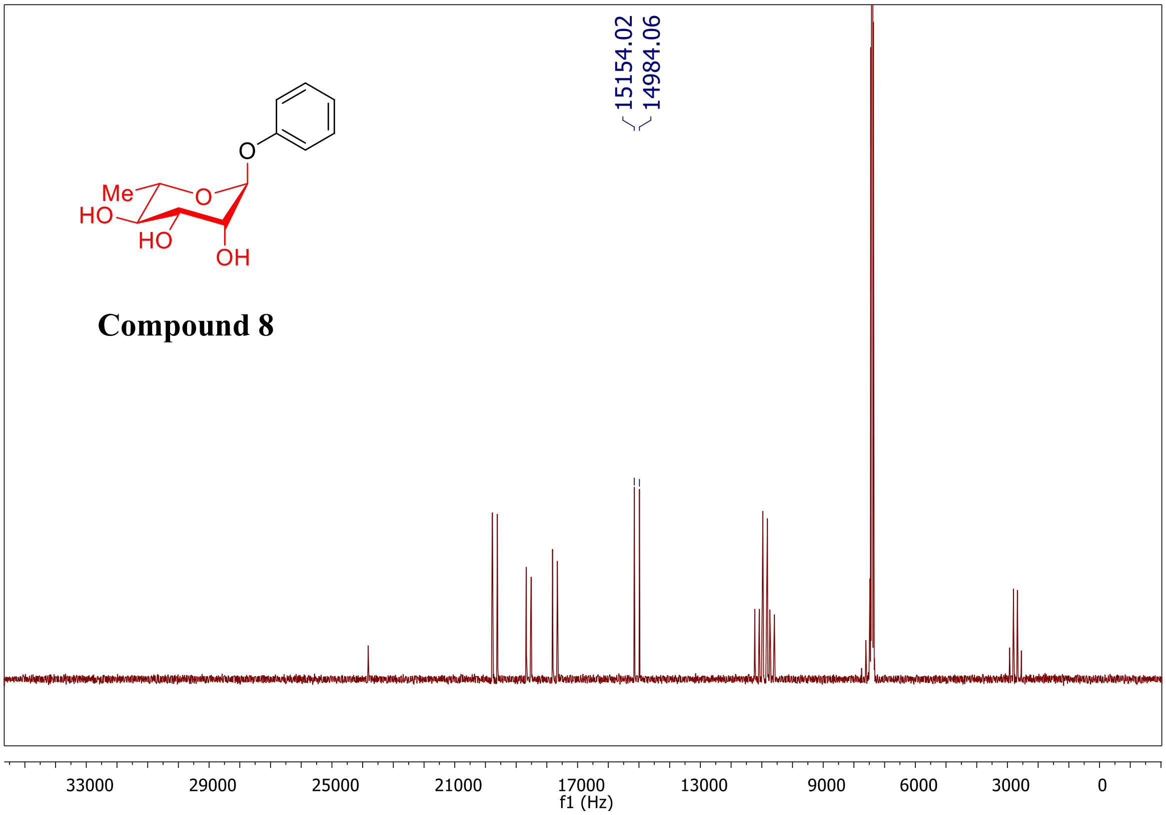
15154.02
14984.06
Me
O
HO
HO
OH
O
Compound 8
33000
29000
25000
21000
17000
13000
9000
6000
3000
0
f1 (Hz)

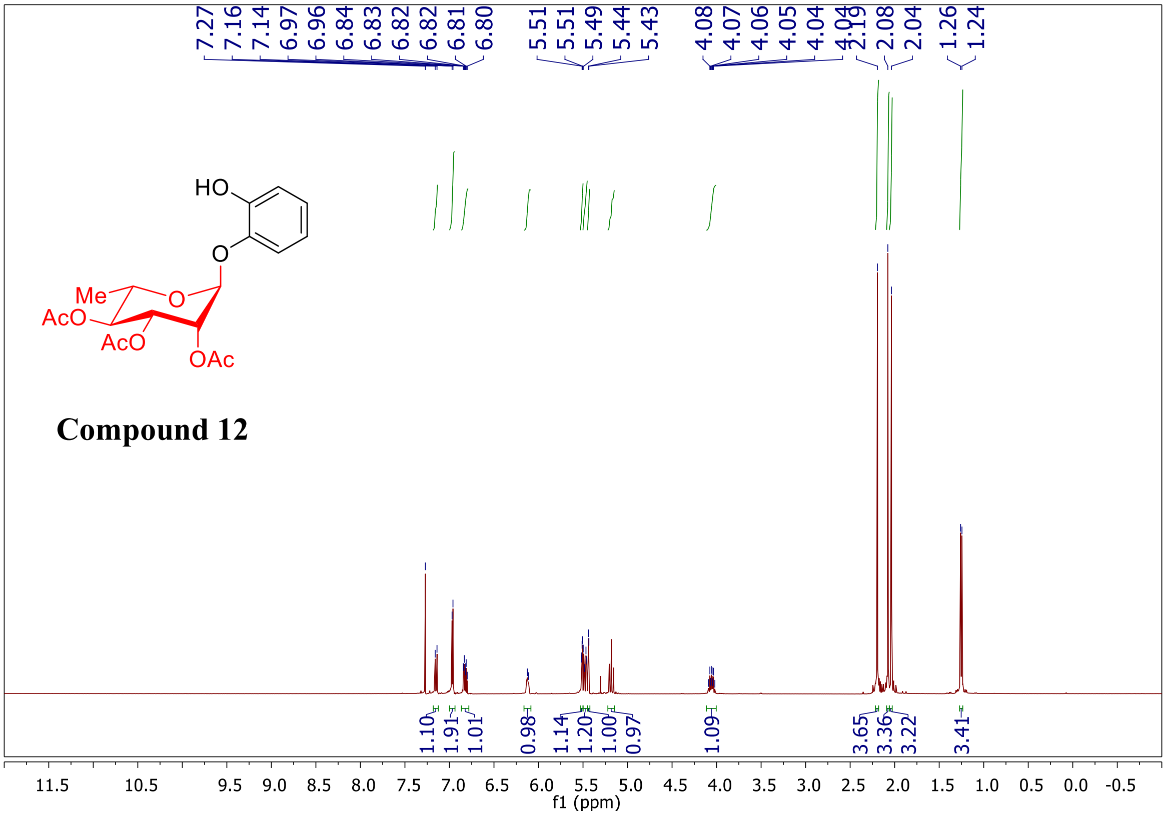
7.27
7.16
7.14
6.97
6.96
6.84
6.83
6.82
6.82
6.81
6.80
5.51
5.51
5.49
5.44
5.43
4.08
4.07
4.06
4.05
4.04
4.04
2.19
2.08
2.04
1.26
1.24
HO
O
Me
O
AcO
AcO
OAc
Compound 12
1.10
1.91
1.01
0.98
1.14
1.20
1.00
0.97
1.09
3.65
3.36
3.22
3.41
11.5
10.5
9.5
9.0
8.5
8.0
7.5
7.0
6.5
6.0
5.5
5.0
4.5
4.0
3.5
3.0
2.5
2.0
1.5
1.0
0.5
0.0
-0.5
f1 (ppm)

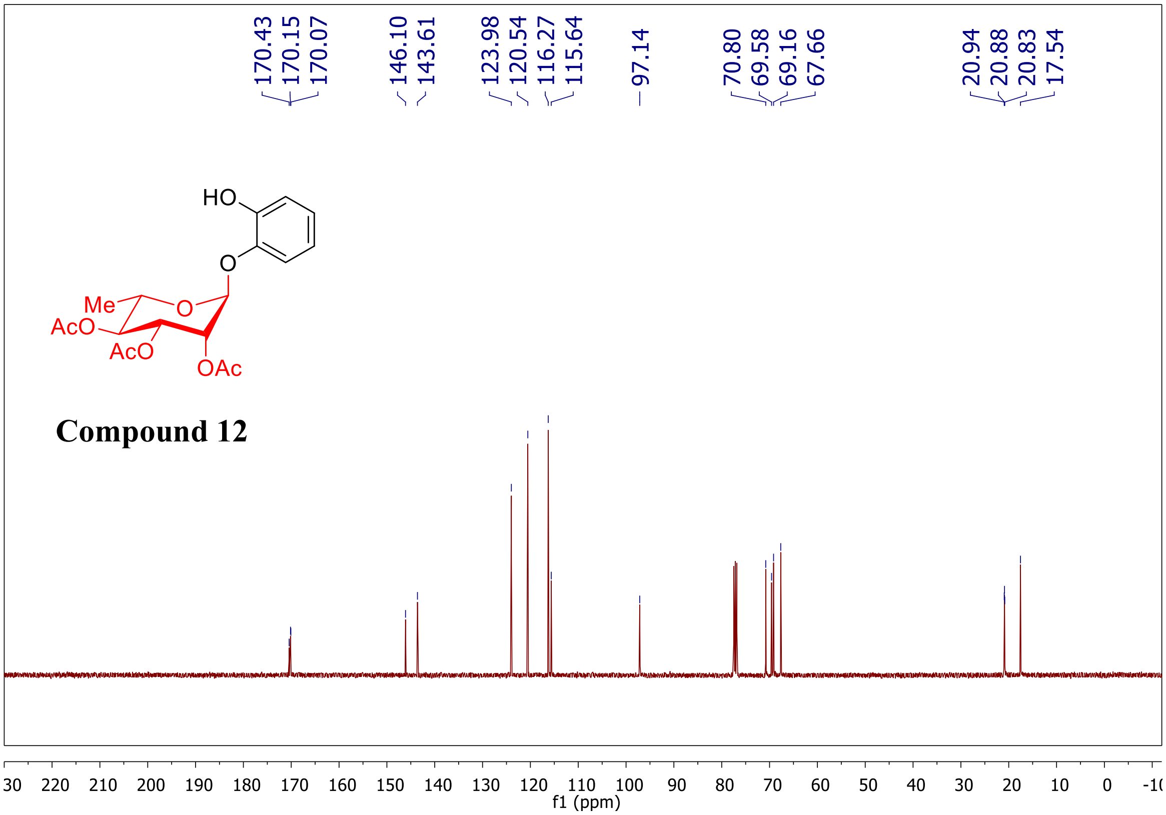

Compound 12

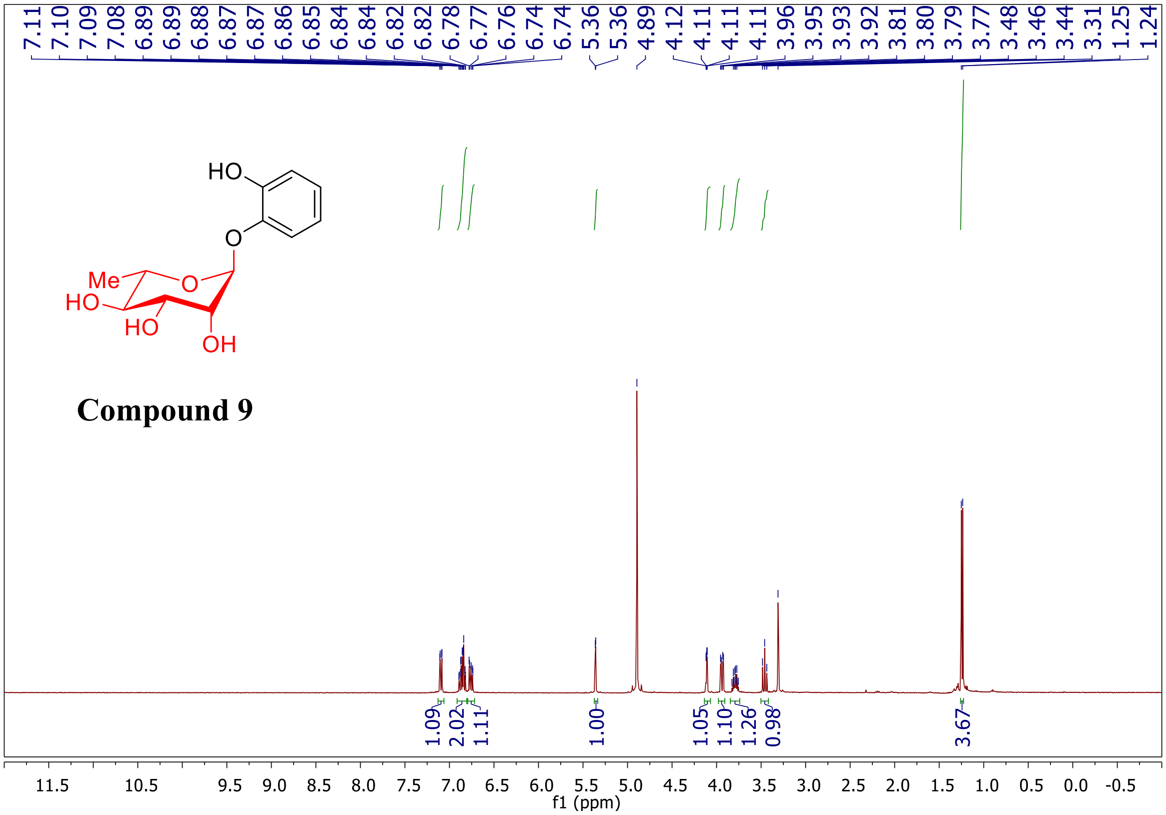

Compound 9

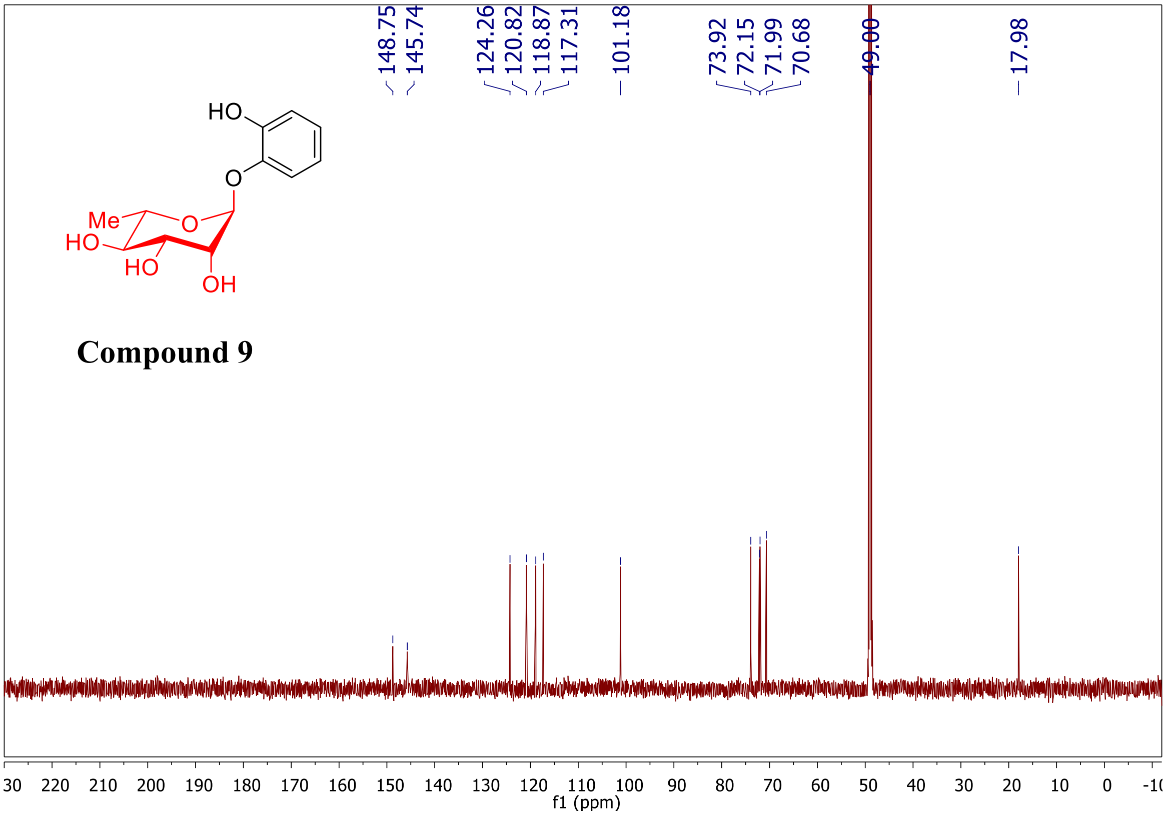
HO
O
Me
O
HO
HO
OH
148.75
145.74
124.26
120.82
118.87
117.31
101.18
73.92
72.15
71.99
70.68
49.00
17.98
f1 (ppm)

Compound 9

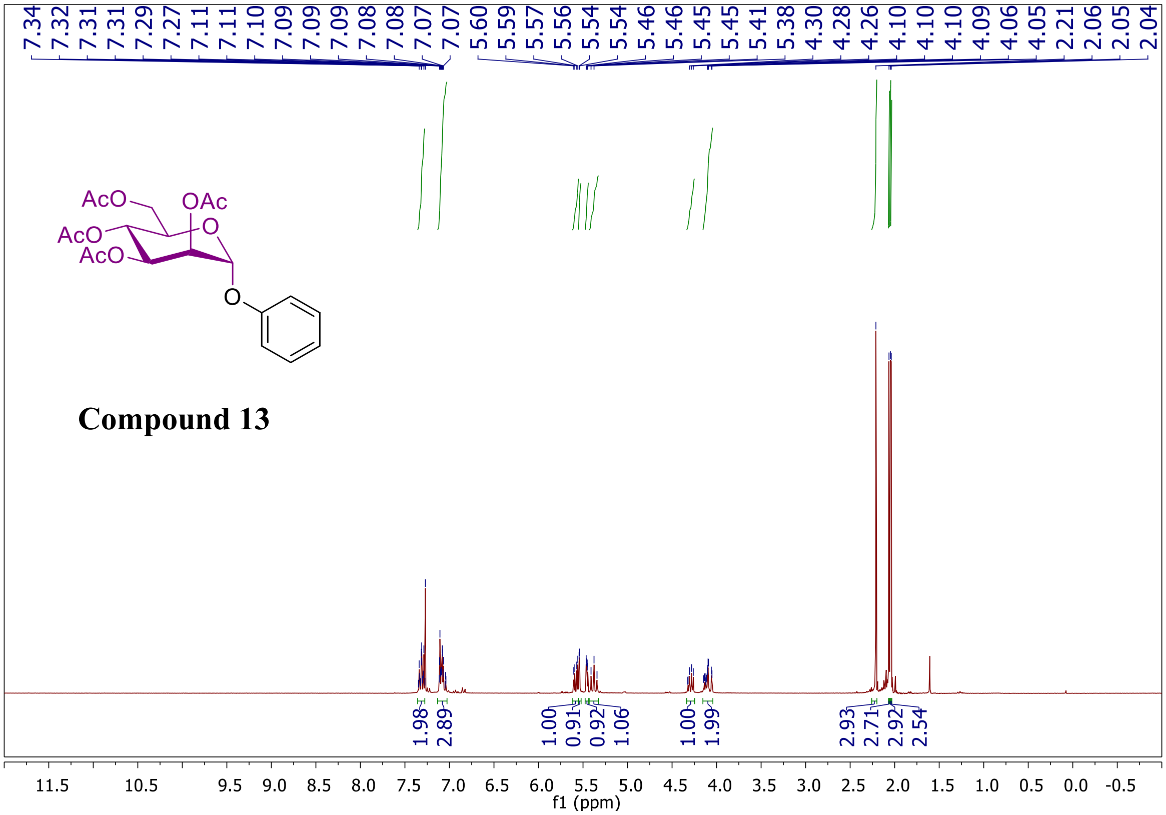
7.34 7.32 7.31 7.31 7.29 7.27 7.11 7.11 7.10 7.09 7.09 7.09 7.08 7.08 7.07 7.07 5.60 5.59 5.57 5.56 5.54 5.54 5.46 5.46 5.45 5.45 5.41 5.38 4.30 4.28 4.26 4.10 4.10 4.10 4.09 4.06 4.05 2.21 2.06 2.05 2.04
AcO
OAc
AcO
AcO
O
O
Compound 13
1.98 2.89 1.00 0.91 0.92 1.06 1.00 1.99 2.93 2.71 2.92 2.54
f1 (ppm)

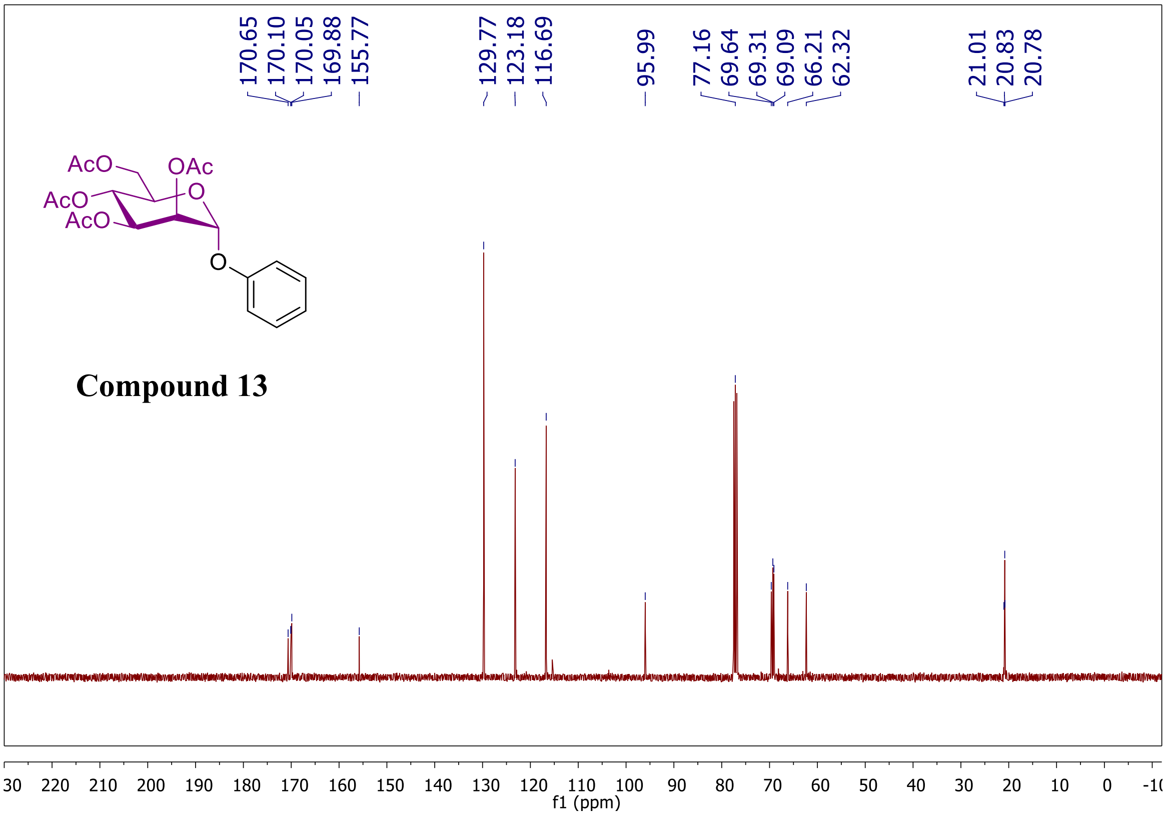
170.65 170.10 170.05 169.88 155.77 129.77 123.18 116.69 95.99 77.16 69.64 69.31 69.09 66.21 62.32 21.01 20.83 20.78
AcO
OAc
AcO
AcO
O
O
Compound 13
f1 (ppm)

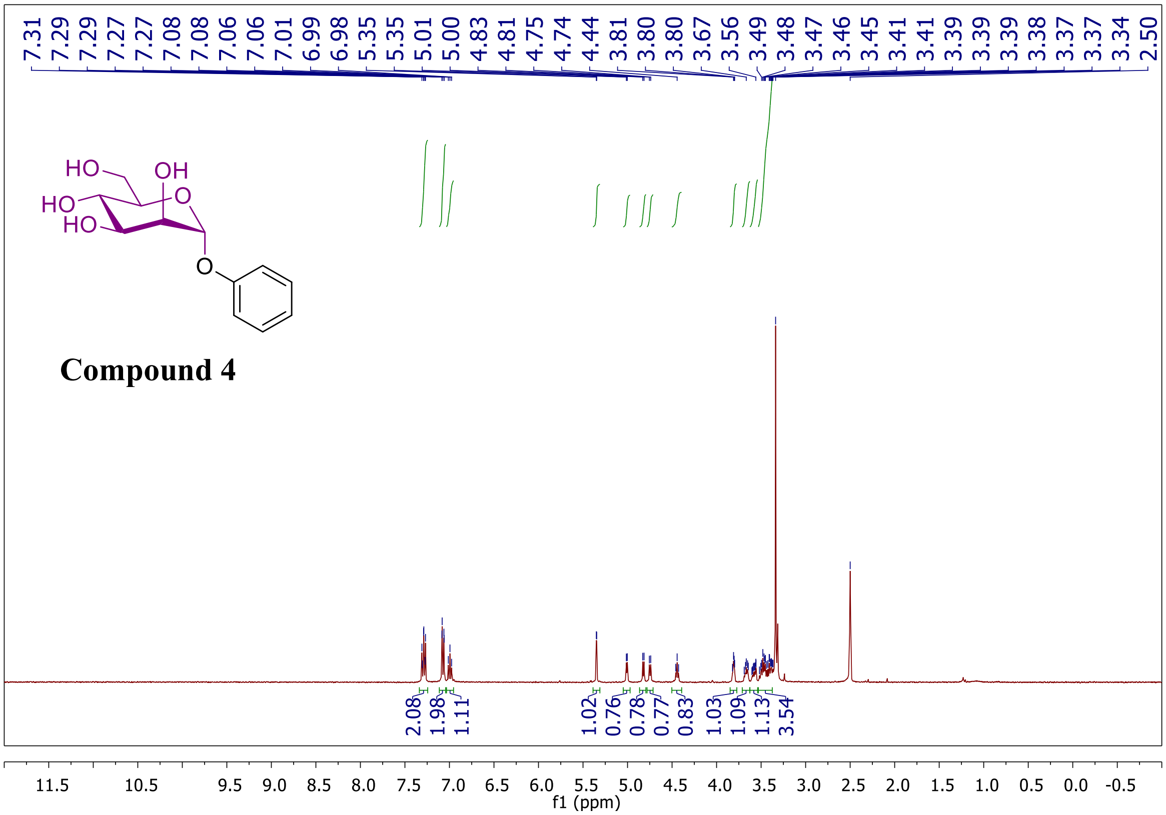
7.31
7.29
7.29
7.27
7.27
7.08
7.08
7.06
7.06
7.01
6.99
6.98
5.35
5.35
5.01
5.00
4.83
4.81
4.75
4.74
4.44
3.81
3.80
3.80
3.67
3.56
3.49
3.48
3.47
3.46
3.45
3.41
3.41
3.39
3.39
3.39
3.38
3.37
3.37
3.34
2.50
HO
OH
O
HO
HO
O
Compound 4
2.08
1.98
1.11
1.02
0.76
0.78
0.77
0.83
1.03
1.09
1.13
3.54
f1 (ppm)

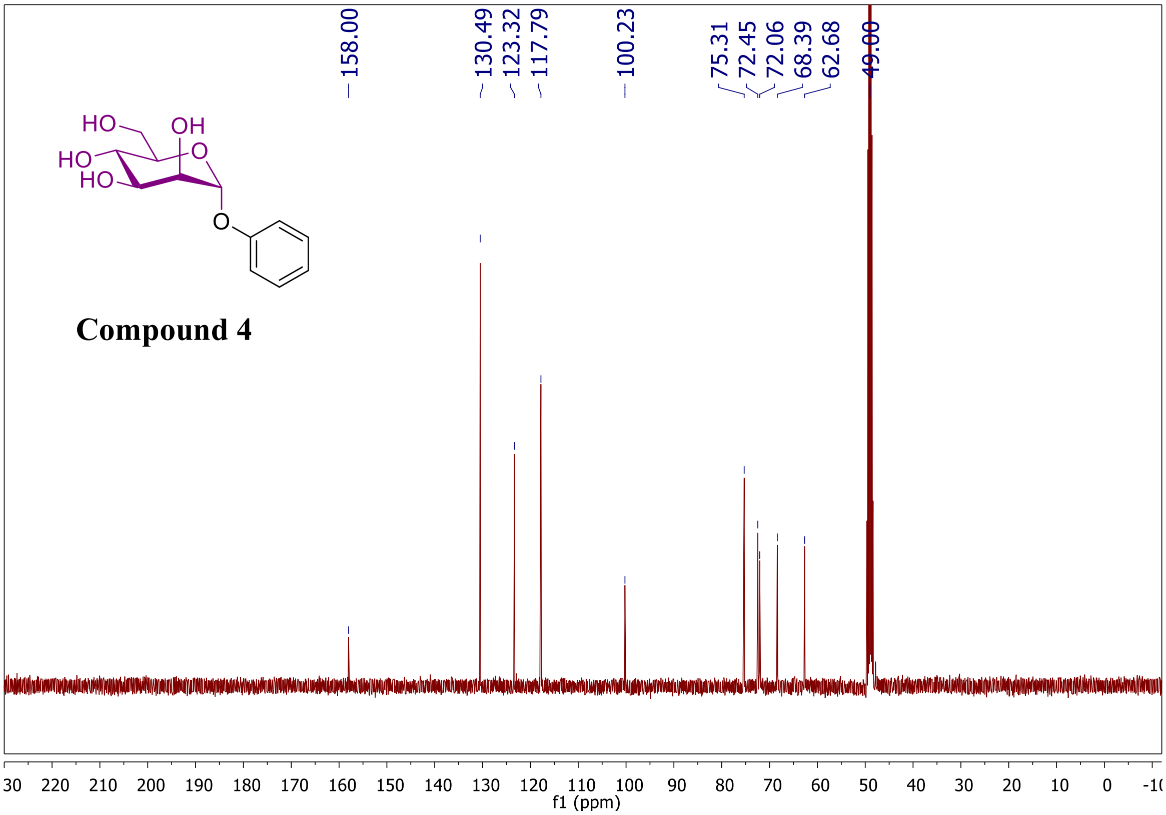
158.00
130.49
123.32
117.79
100.23
75.31
72.45
72.06
68.39
62.68
49.00
HO
OH
O
HO
HO
O
Compound 4
f1 (ppm)

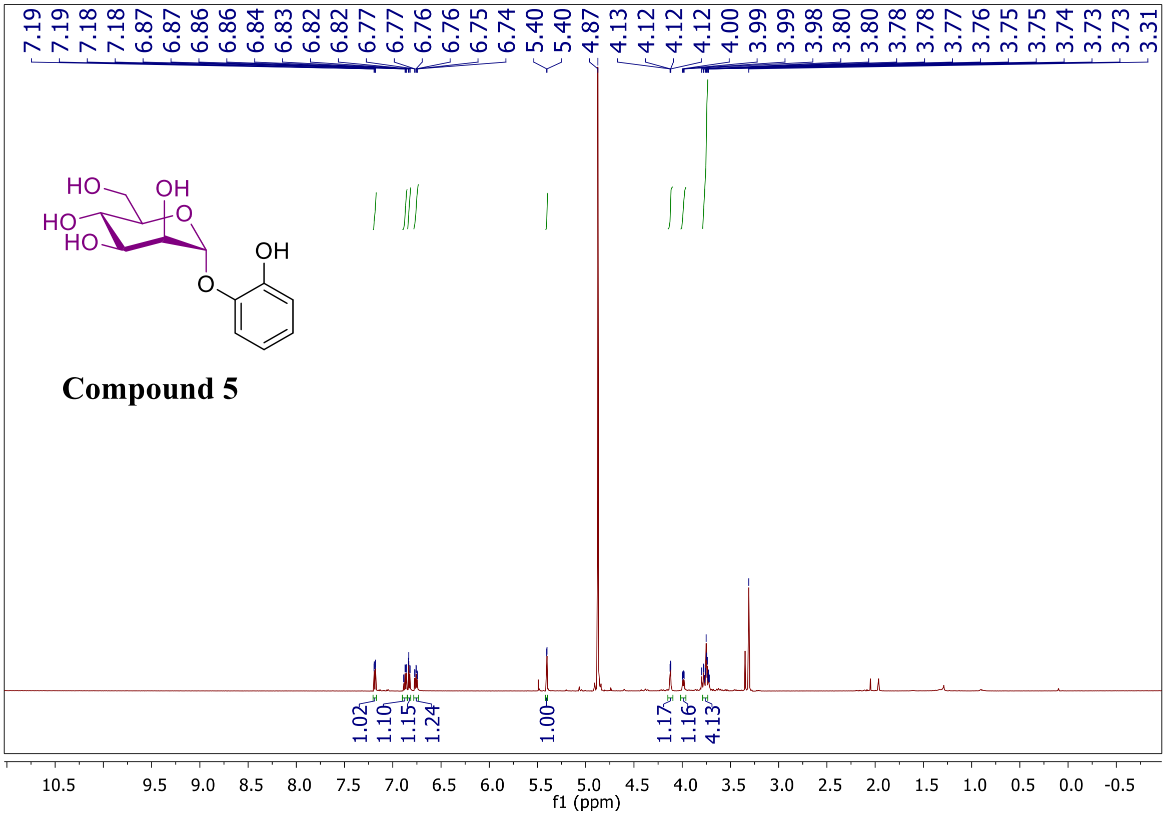
7.19 7.19 7.18 7.18 6.87 6.87 6.86 6.86 6.84 6.83 6.82 6.82 6.77 6.77 6.76 6.76 6.75 6.74 5.40 5.40 4.87 4.13 4.12 4.12 4.12 4.00 3.99 3.99 3.98 3.80 3.80 3.78 3.78 3.77 3.76 3.75 3.75 3.74 3.73 3.73 3.31
HO
OH
O
HO
HO
OH
O
Compound 5
1.02
1.10
1.15
1.24
1.00
1.17
1.16
4.13
10.5 9.5 9.0 8.5 8.0 7.5 7.0 6.5 6.0 5.5 5.0 4.5 4.0 3.5 3.0 2.5 2.0 1.5 1.0 0.5 0.0 -0.5
f1 (ppm)

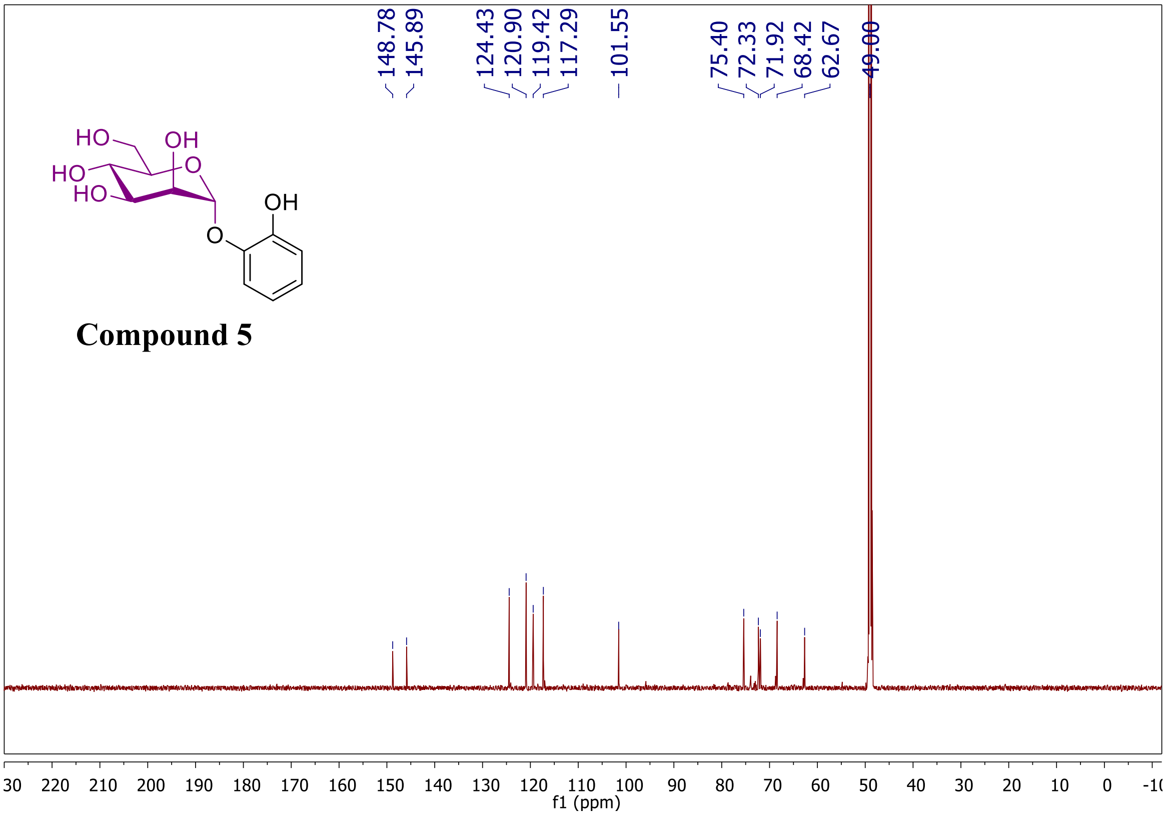
148.78 145.89 124.43 120.90 119.42 117.29 101.55 75.40 72.33 71.92 68.42 62.67 49.00
HO
OH
O
HO
HO
OH
O
Compound 5
220 210 200 190 180 170 160 150 140 130 120 110 100 90 80 70 60 50 40 30 20 10 0
f1 (ppm)

^{13}C-^{1}H coupled NMR (anomeric $^{1}J_{CH}$ = 171.46 Hz)

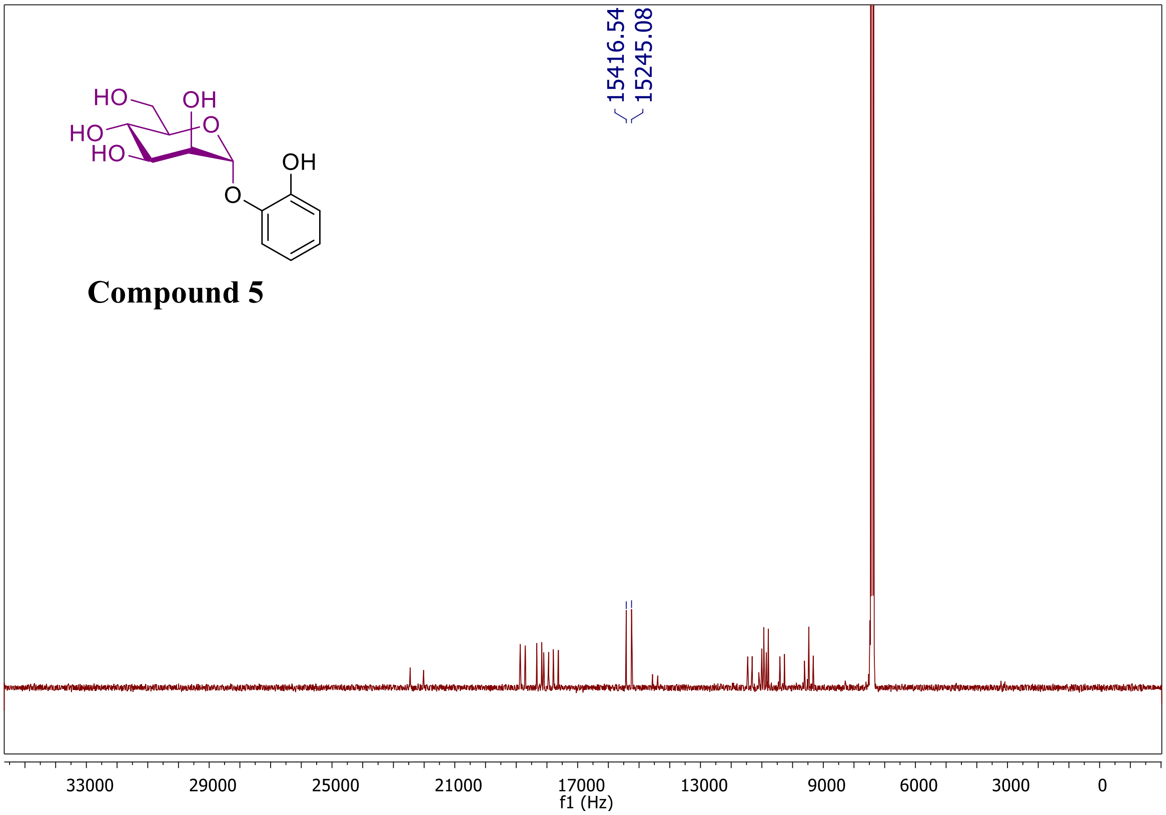

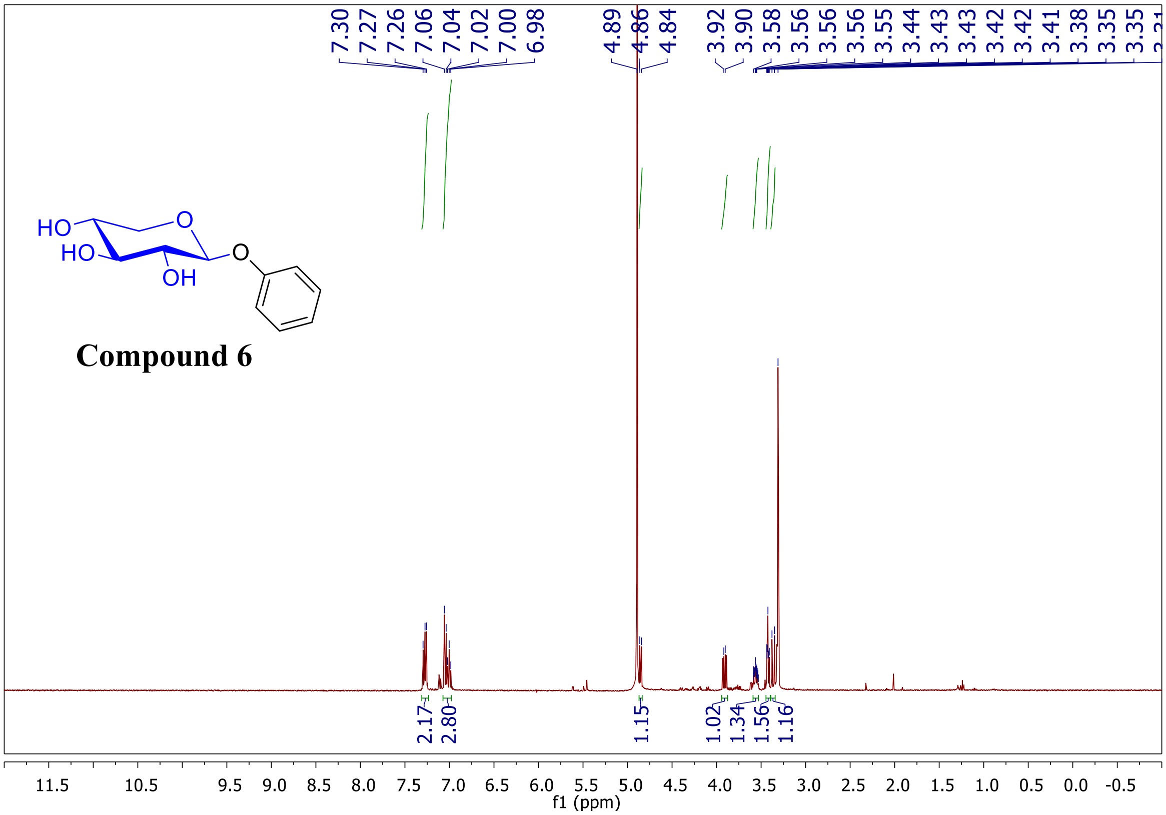

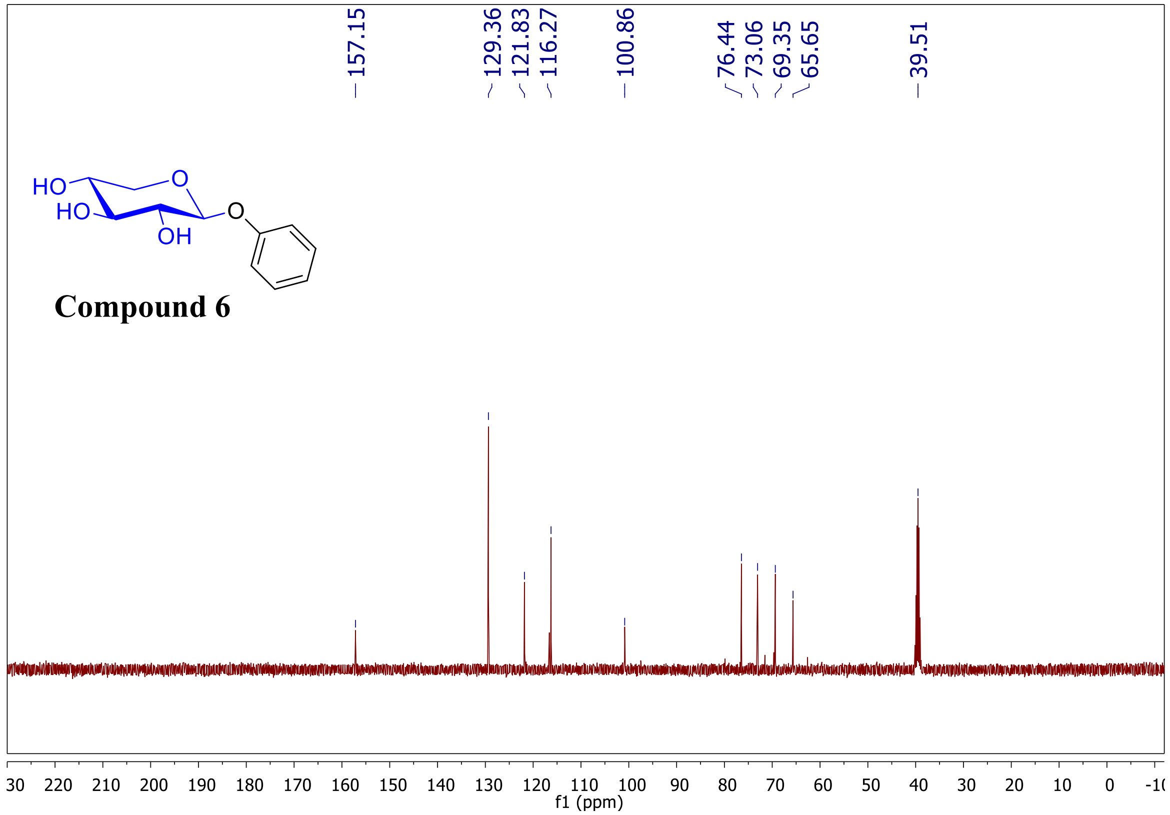
157.15
129.36
121.83
116.27
100.86
76.44
73.06
69.35
65.65
39.51
HO
HO
O
OH
O
Compound 6
30 220 210 200 190 180 170 160 150 140 130 120 110 100 90 80 70 60 50 40 30 20 10 0
f1 (ppm)

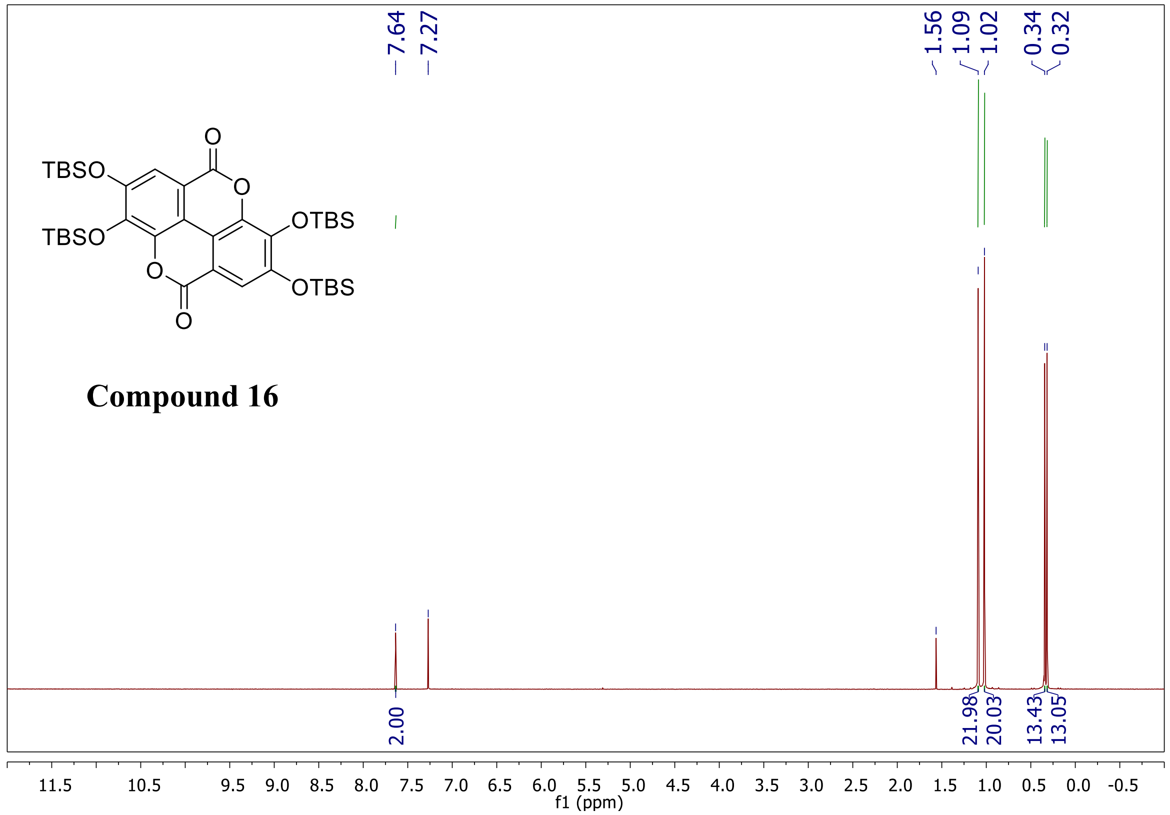
7.64
7.27
1.56
1.09
1.02
0.34
0.32
TBSO
TBSO
OTBS
OTBS
O
O
O
O
Compound 16
2.00
21.98
20.03
13.43
13.05
11.5 10.5 9.5 9.0 8.5 8.0 7.5 7.0 6.5 6.0 5.5 5.0 4.5 4.0 3.5 3.0 2.5 2.0 1.5 1.0 0.5 0.0 -0.5
f1 (ppm)

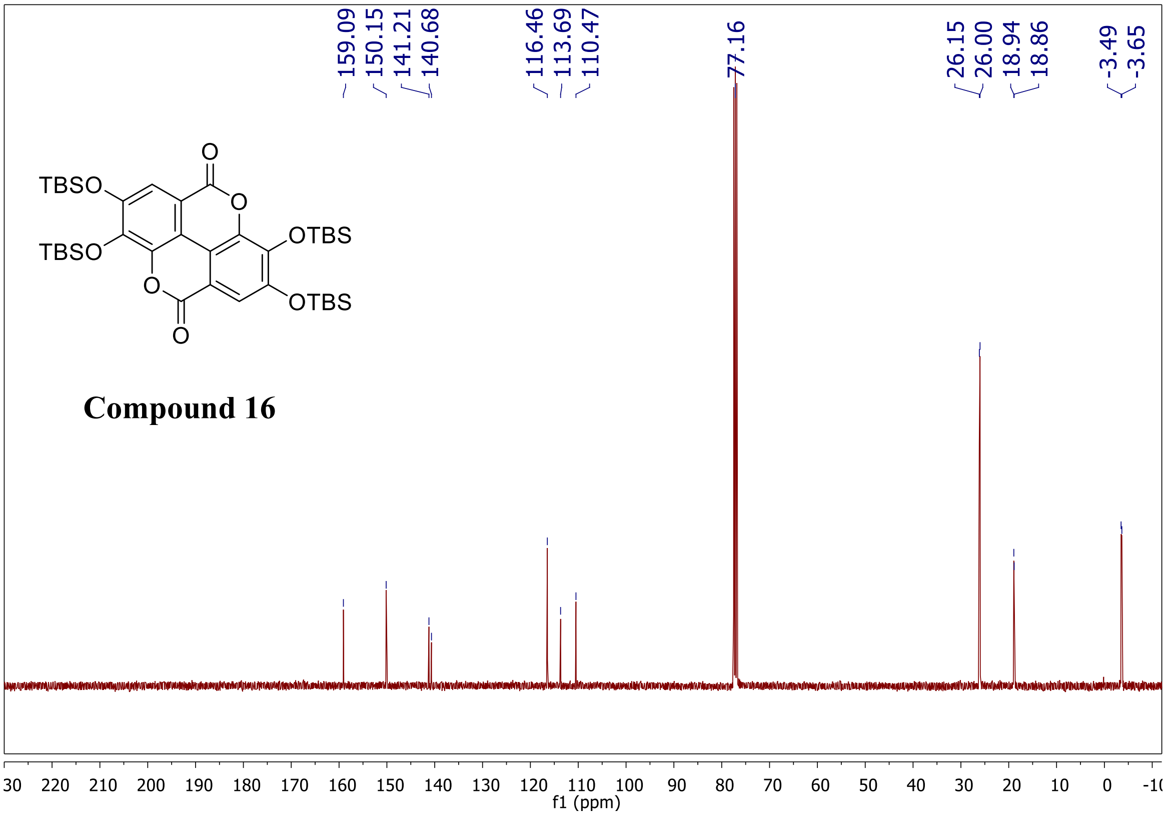
159.09
150.15
141.21
140.68
116.46
113.69
110.47
77.16
26.15
26.00
18.94
18.86
-3.49
-3.65
TBSO
TBSO
OTBS
OTBS
O
O
O
O
f1 (ppm)

Compound 16

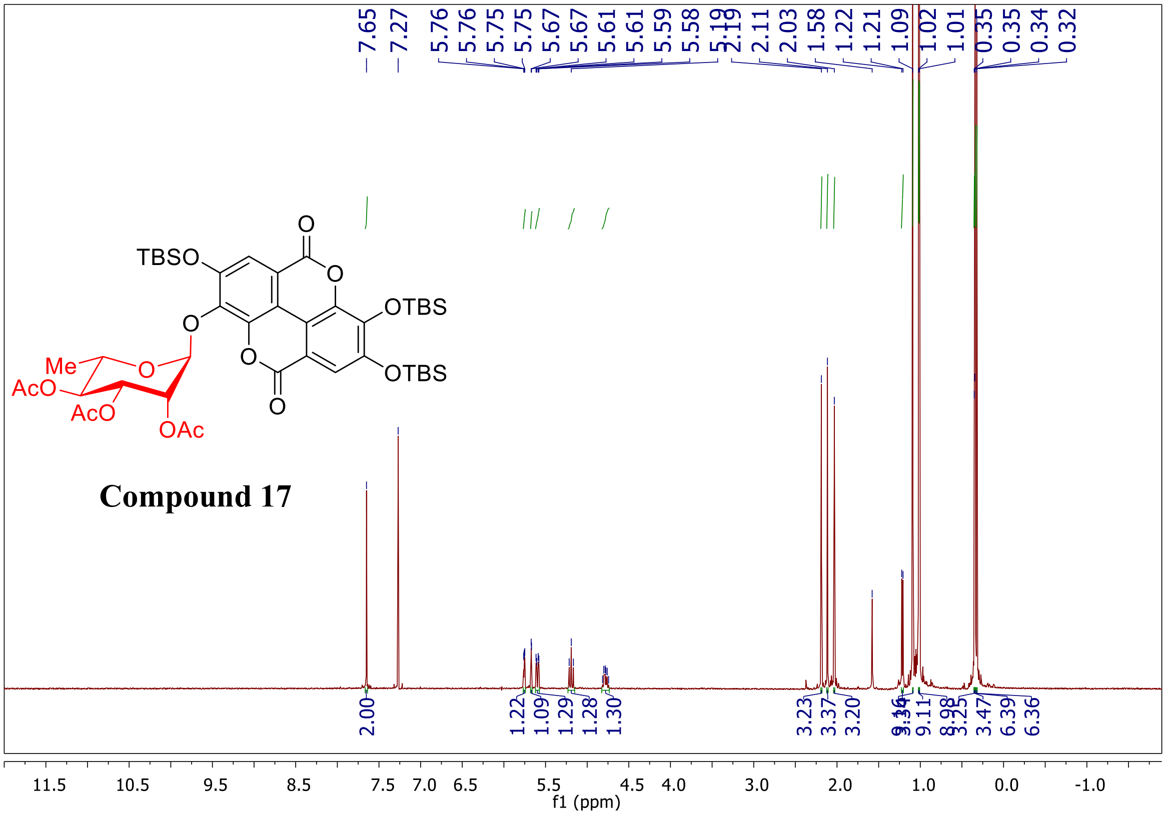
7.65
7.27
5.76
5.76
5.75
5.75
5.67
5.67
5.61
5.61
5.59
5.58
5.19
2.19
2.11
2.03
1.58
1.22
1.21
1.09
1.02
1.01
0.35
0.35
0.34
0.32
TBSO
O
O
OTBS
OTBS
Me
AcO
AcO
OAc
O
Compound 17
2.00
1.22
1.09
1.29
1.28
1.30
3.23
3.37
3.20
9.16
3.34
9.11
8.98
3.25
3.47
6.39
6.36
11.5
10.5
9.5
8.5
7.5
7.0
6.5
5.5
4.5
4.0
3.0
2.0
1.0
0.0
-1.0
f1 (ppm)

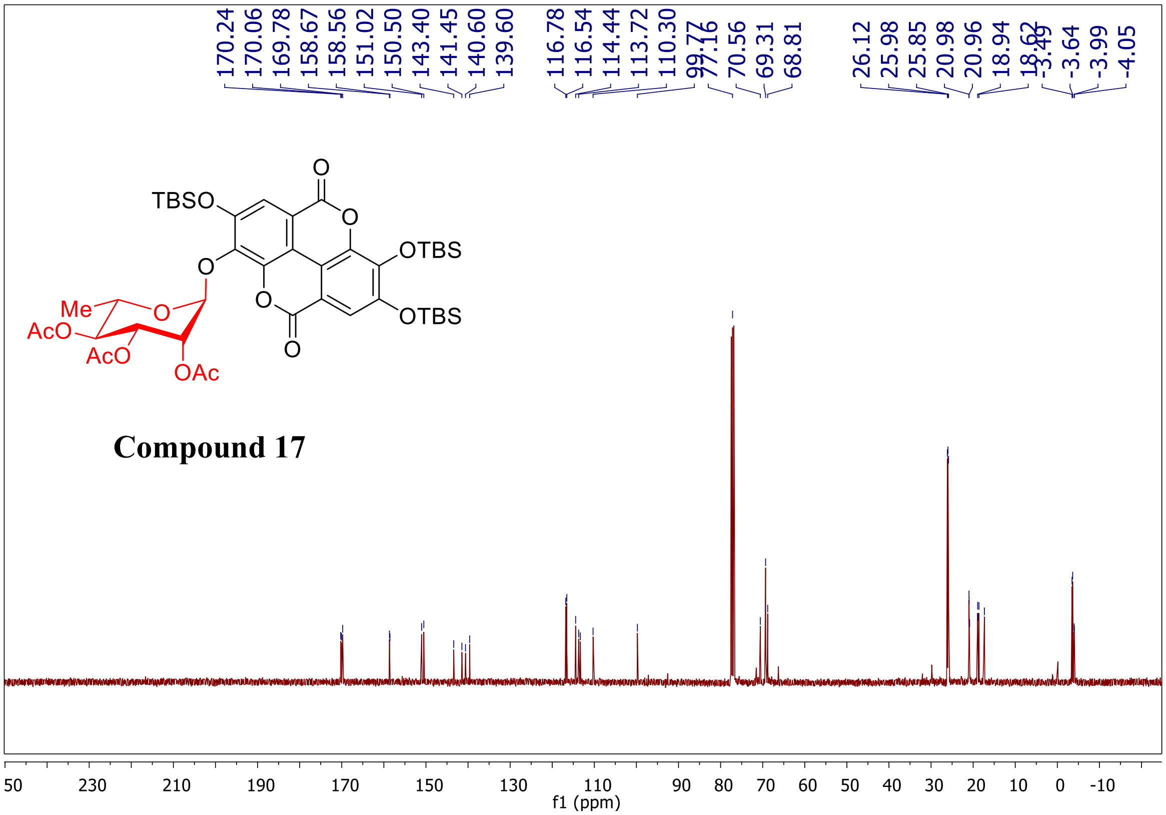
170.24
170.06
169.78
158.67
158.56
151.02
150.50
143.40
141.45
140.60
139.60
116.78
116.54
114.44
113.72
110.30
99.77
77.16
70.56
69.31
68.81
26.12
25.98
25.85
20.98
20.96
18.94
18.62
-3.49
-3.64
-3.99
-4.05
TBSO
O
O
OTBS
OTBS
Me
AcO
AcO
OAc
O
Compound 17
50
230
210
190
170
150
130
110
90
80
70
60
50
40
30
20
10
0
-10
f1 (ppm)

^{13}C-^{1}H coupled NMR (anomeric $^{1}J_{CH}$ = 177.75 Hz)

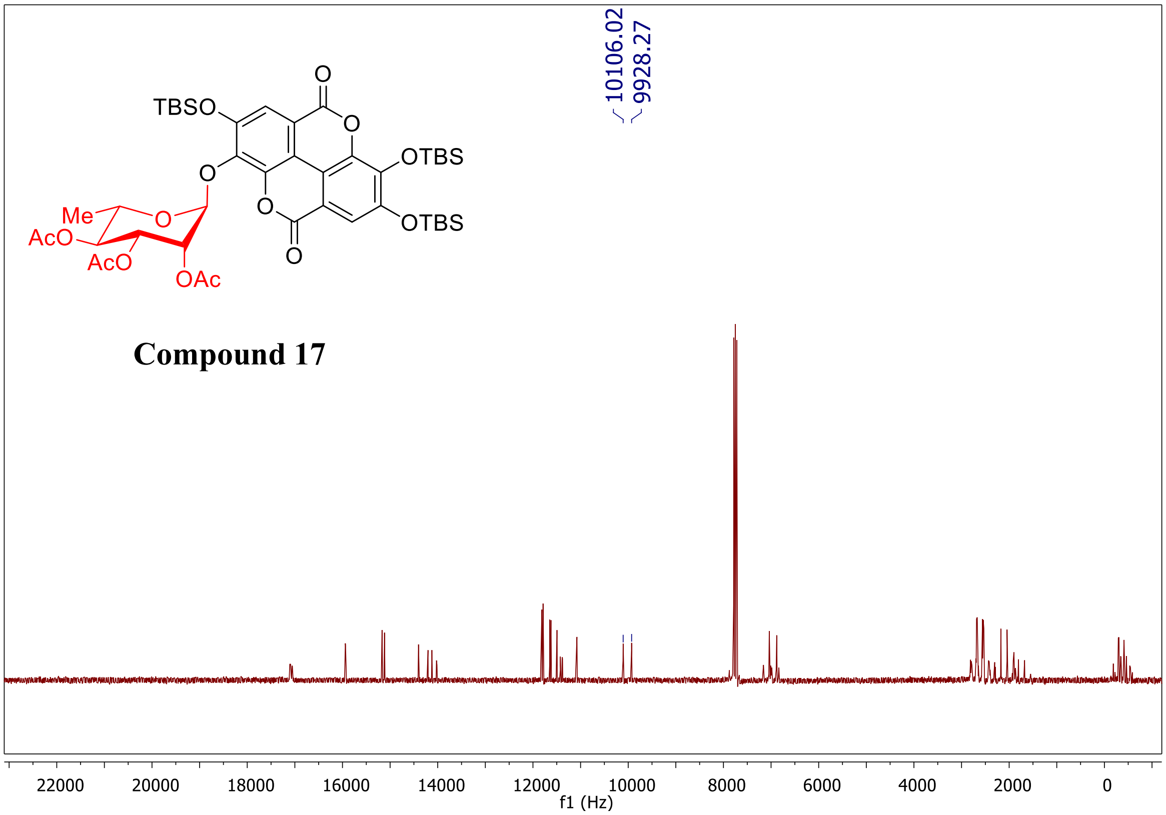

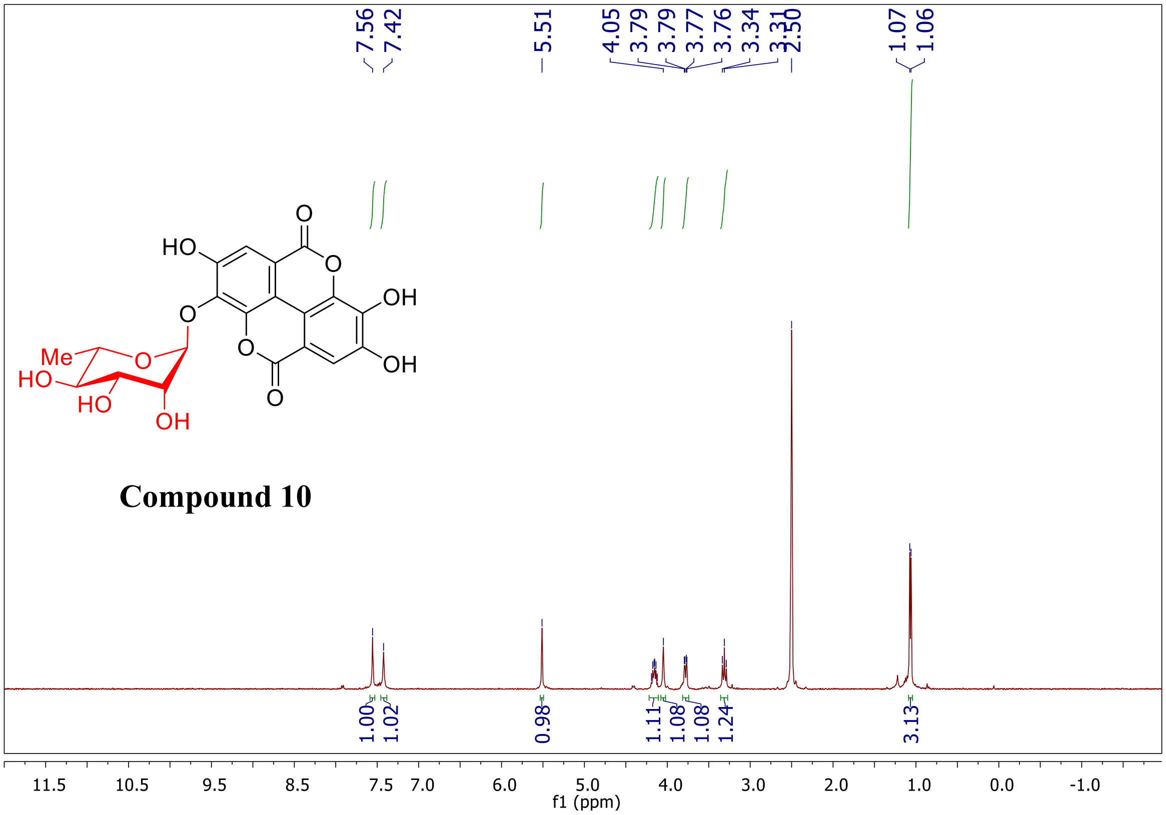
7.56
7.42
5.51
4.05
3.79
3.79
3.77
3.76
3.34
3.31
2.50
1.07
1.06
HO
O
OH
OH
O
Me
HO
HO
OH
Compound 10
1.00
1.02
0.98
1.11
1.08
1.08
1.24
3.13
11.5 10.5 9.5 8.5 7.5 7.0 6.0 5.0 4.0 3.0 2.0 1.0 0.0 -1.0
f1 (ppm)

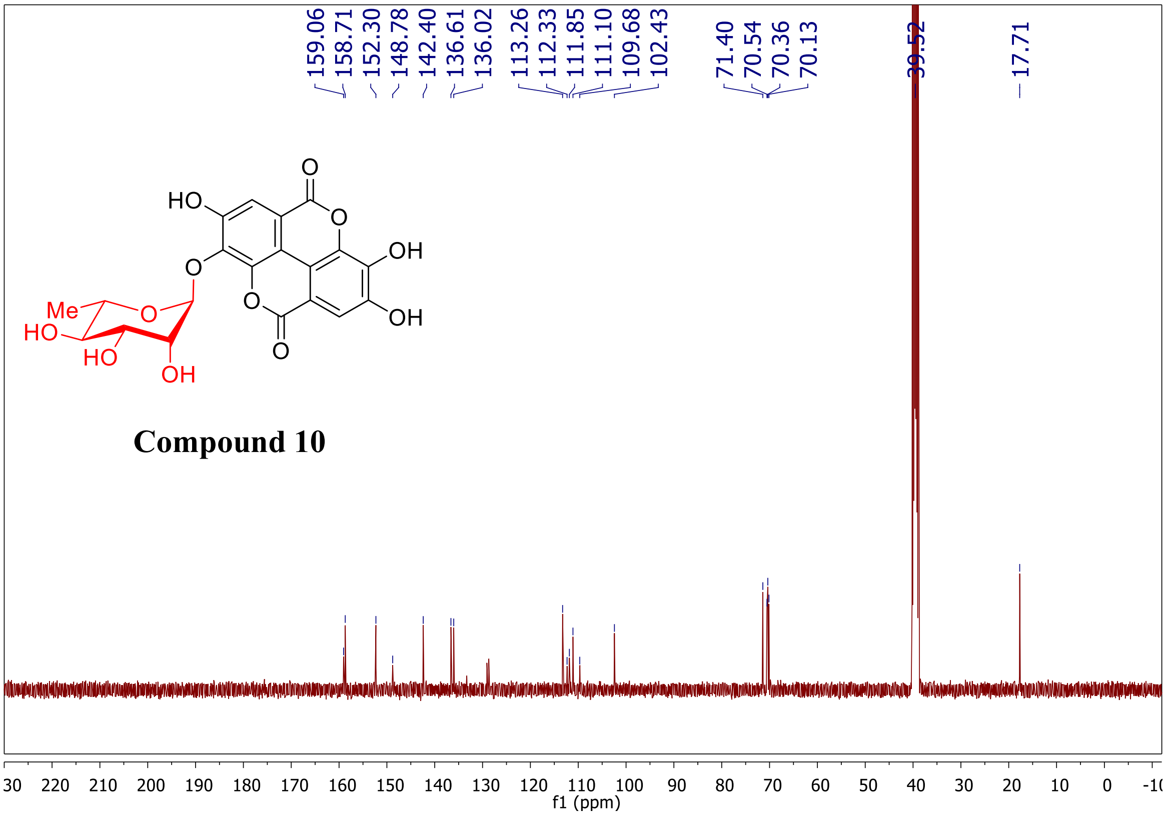
159.06
158.71
152.30
148.78
142.40
136.61
136.02
113.26
112.33
111.85
111.10
109.68
102.43
71.40
70.54
70.36
70.13
39.52
17.71
HO
O
OH
OH
O
Me
HO
HO
OH
Compound 10
30 220 210 200 190 180 170 160 150 140 130 120 110 100 90 80 70 60 50 40 30 20 10 0
f1 (ppm)

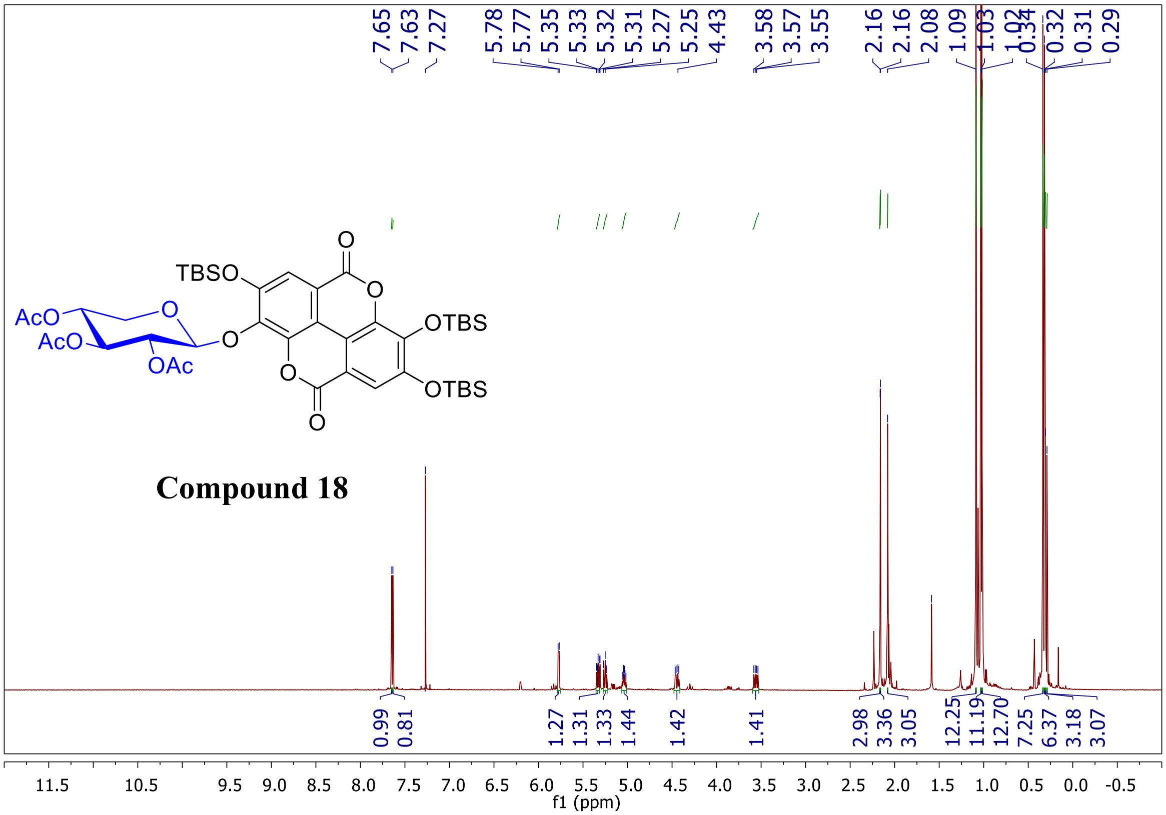

Compound 18

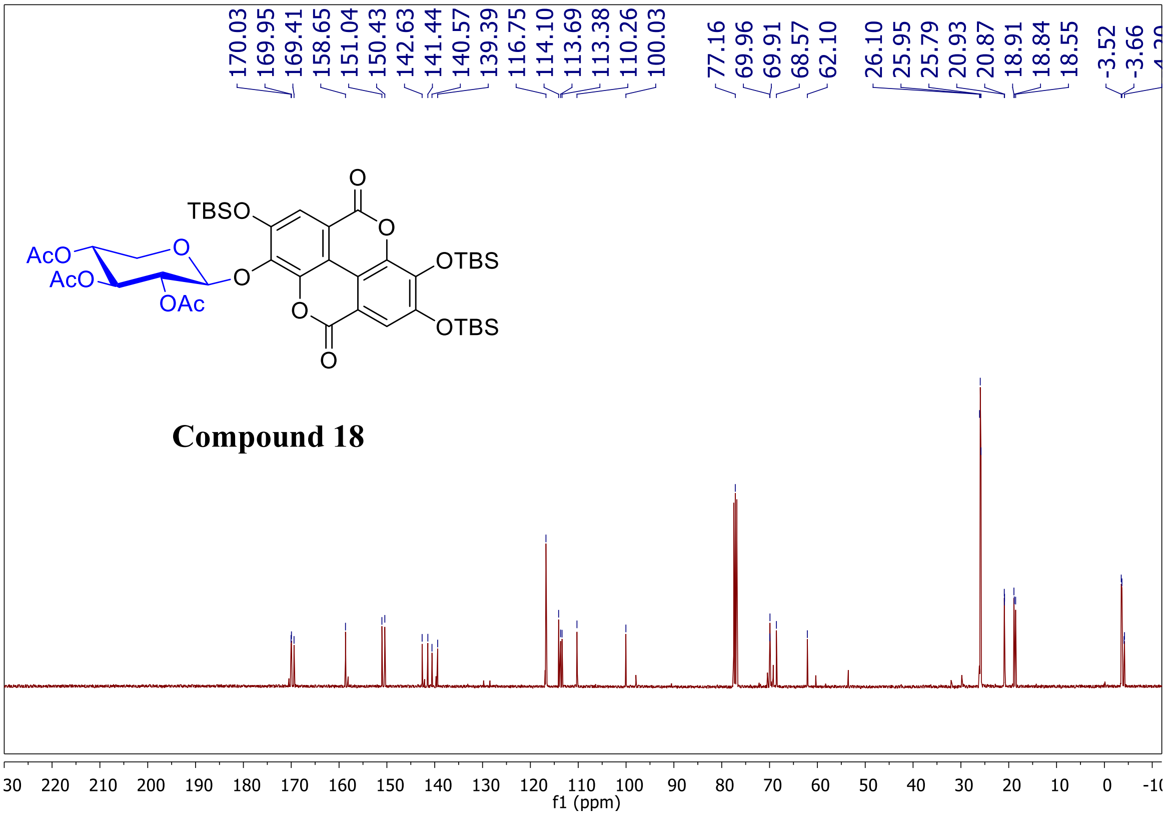

Compound 18

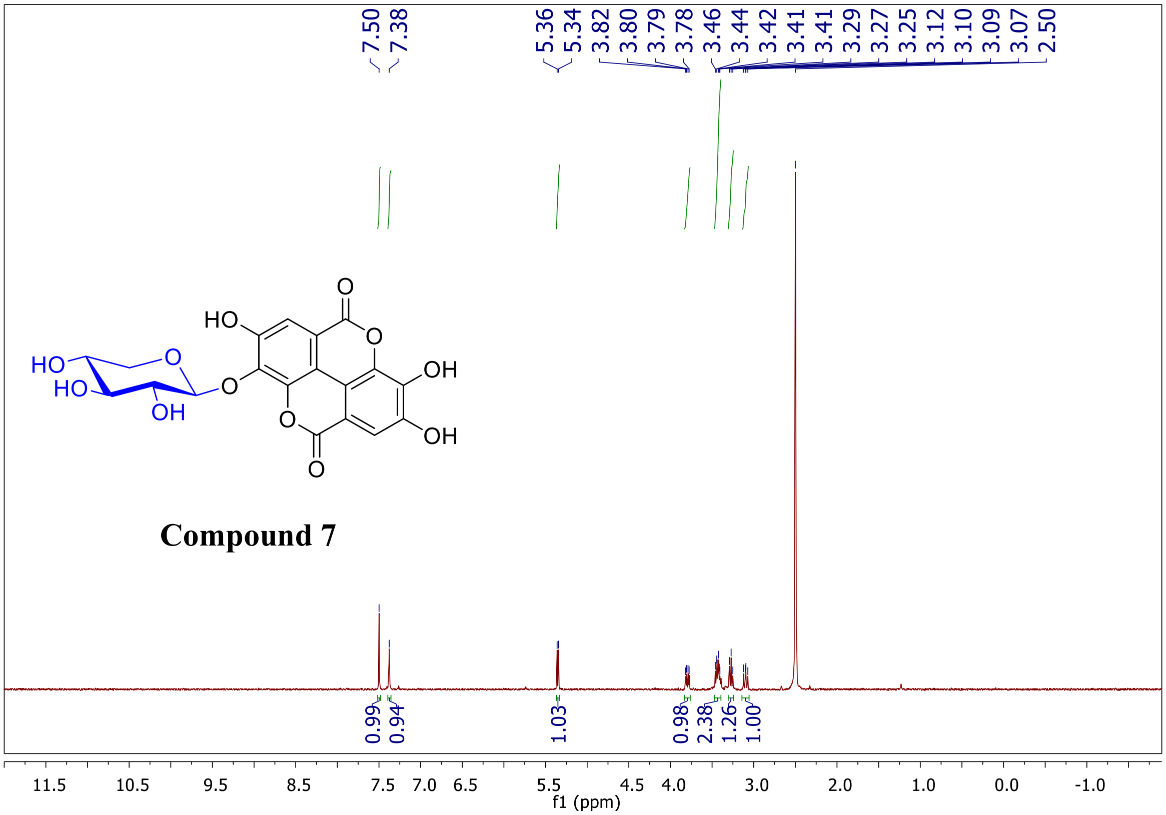
7.50
7.38
5.36
5.34
3.82
3.80
3.79
3.78
3.46
3.44
3.42
3.41
3.41
3.29
3.27
3.25
3.12
3.10
3.09
3.07
2.50
0.99
0.94
1.03
0.98
2.38
1.26
1.00
HO
HO
HO
OH
O
O
O
O
O
O
O
OH
OH
Compound 7
11.5
10.5
9.5
8.5
7.5
7.0
6.5
5.5
4.5
4.0
3.0
2.0
1.0
0.0
-1.0
f1 (ppm)

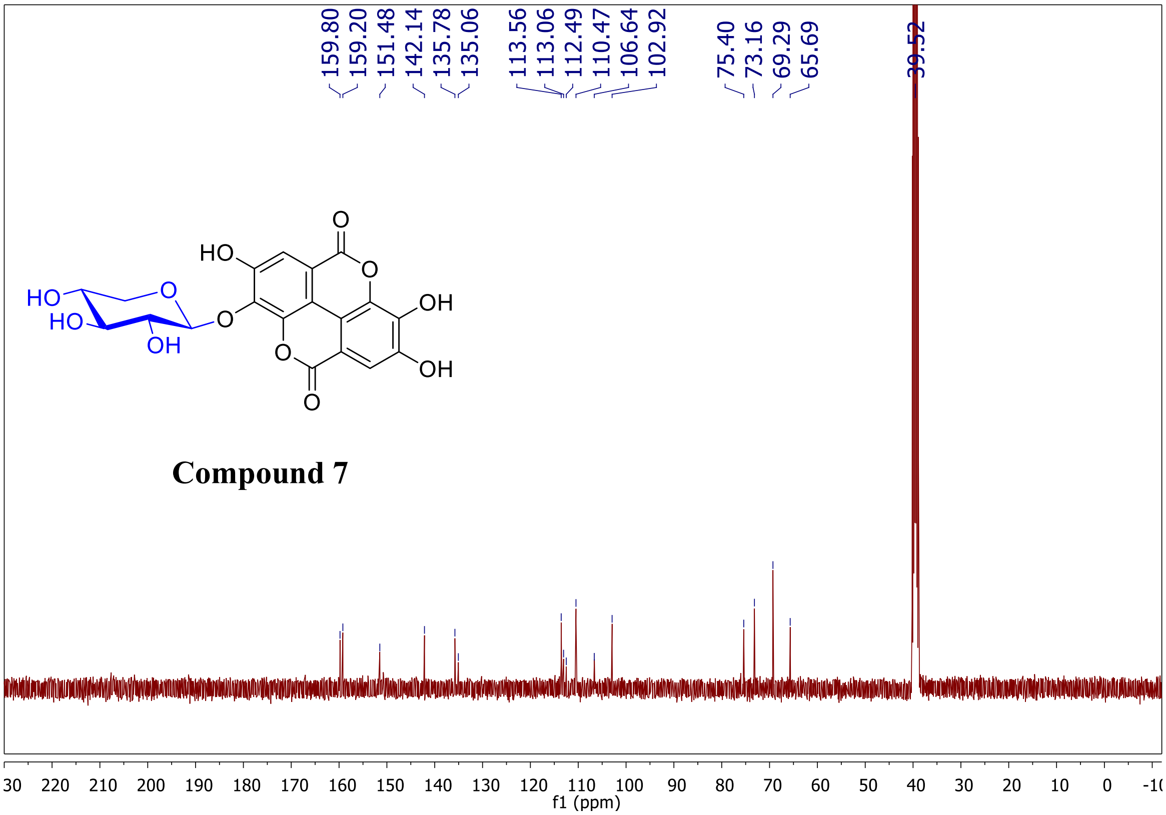
159.80
159.20
151.48
142.14
135.78
135.06
113.56
113.06
112.49
110.47
106.64
102.92
75.40
73.16
69.29
65.69
39.52
HO
HO
HO
OH
O
O
O
O
O
O
O
OH
OH
Compound 7
30
220
210
200
190
180
170
160
150
140
130
120
110
100
90
80
70
60
50
40
30
20
10
0
f1 (ppm)
